# Centromeric binding and activity of Protein Phosphatase 4

**DOI:** 10.1038/ncomms6894

**Published:** 2015-01-06

**Authors:** Zoltan Lipinszki, Stephane Lefevre, Matthew S. Savoian, Martin R. Singleton, David M. Glover, Marcin R. Przewloka

**Affiliations:** 1 Department of Genetics, University of Cambridge, Downing Street, Cambridge CB2 3EH, UK; 2 Macromolecular Structure and Function Laboratory, Cancer Research UK, London Research Institute, London WC2A 3LY, UK

## Abstract

The cell division cycle requires tight coupling between protein phosphorylation and dephosphorylation. However, understanding the cell cycle roles of multimeric protein phosphatases has been limited by the lack of knowledge of how their diverse regulatory subunits target highly conserved catalytic subunits to their sites of action. Phosphoprotein phosphatase 4 (PP4) has been recently shown to participate in the regulation of cell cycle progression. We now find that the EVH1 domain of the regulatory subunit 3 of Drosophila PP4, Falafel (Flfl), directly interacts with the centromeric protein C (CENP-C). Unlike other EVH1 domains that interact with proline-rich ligands, the crystal structure of the Flfl amino-terminal EVH1 domain bound to a CENP-C peptide reveals a new target-recognition mode for the phosphatase subunit. We also show that binding of Flfl to CENP-C is required to bring PP4 activity to centromeres to maintain CENP-C and attached core kinetochore proteins at chromosomes during mitosis.

## Abstract (web summary)

Many cellular processes, including the cell division cycle, require concerted action of protein kinases and phosphatases. Here Lipinszki *et al*. present a crystal structure of the *Drosophila* phosphoprotein phosphatase 4 subunit, Falafel, in complex with the centromeric protein CENP-C, and reveal a new recognition mode for this phosphatase.

## Graphical abstract

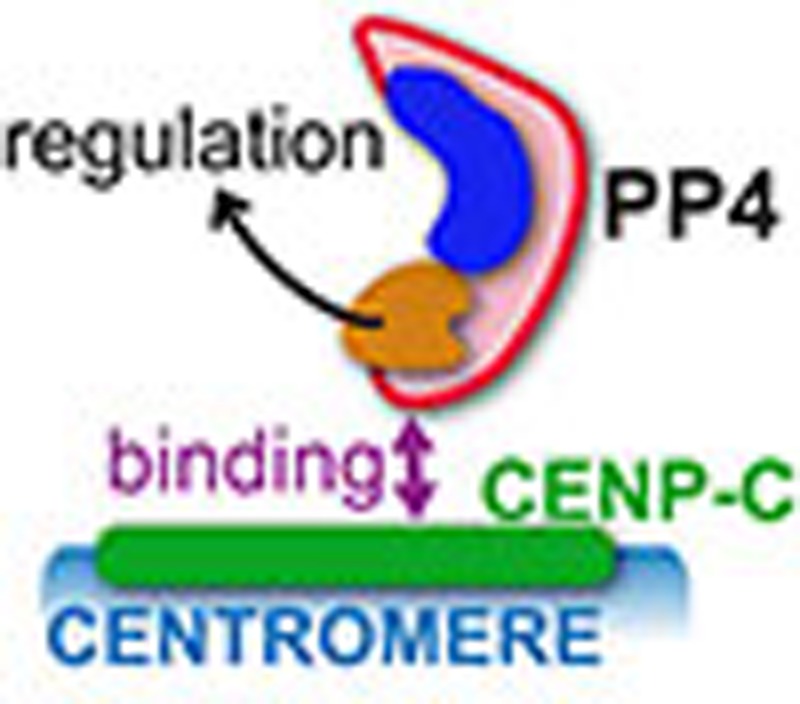

Reversible protein phosphorylation is a highly conserved regulatory mechanism that is crucial for orchestrating fundamental physiological processes such as cell division. Several members of the large phosphoprotein phosphatase (PPP) family of Ser/Thr phosphatases (the PP1-related family; the PP2A family of PP2A, PP4 and PP6; PP2B and PP5) have been linked to mitotic regulation^1^. However, our understanding of these enzymes is largely incomplete, in part because of the highly conserved nature of the catalytic subunits that rely on a wide range of targeting subunits to provide specificity. Much of our insight is based on PP1 and PP2A (refs 1, 2), with little data available on the substrates and regulation of the other PPP family members.

Recent studies revealed that protein phosphatase 4 (PP4), a ubiquitous and essential PPP, regulates a variety of cellular processes^3^, including chromatin biology^4^, DNA repair^5^,^6^,^7^ and cell cycle progression^8^,^9^,^10^. Surprisingly, very few PP4 substrates have been identified so far. Like its family member PP2A (ref. 1), PP4 often functions as a heterotrimeric complex^3^,^11^ consisting of one evolutionarily conserved catalytic subunit (PP4c) that associates with two types of regulatory subunits: a structural protein, PP4R2 (R2, and R1 in mammals), and a regulatory 3 (R3) subunit, PP4R3 (platinum sensitivity 2 (Psy2) in yeast^12^,^13^, Falafel (Flfl)^14^ in Drosophila, suppressor of MEK1 (SMEK1; termed R3α^11^) and SMEK2 (termed R3β^11^), the two known isoforms in human). The major form of PP4, conserved from yeast to human cells, comprises PP4c in complex with R2 and R3. However, in metazoans PP4c can also associate with other ancillary proteins (for example, R4, HDAC3, α4 or Gemin4 (see refs 3, 11 and 15)). Accordingly, PP4c has been found to be a component of several mutually exclusive complexes including the PP4c-R2-R3 (R3α or β) heterotrimer, but also PP4c-R1-HDAC3, PP4c-R4 or PP4c-α4, that presumably have distinct substrates and biological roles. Current models propose that PP4 functions through the modular activity of its component subunits. But how these regulators co-operate in substrate selectivity or sub-cellular localization and stabilization of the holoenzyme is poorly understood.

A recent study, for example, indicates that the PP4c–R3β complex does not require other regulatory subunits (for example, R2 or R1) to dephosphorylate 53BP1 in DNA repair processes^6^. In contrast, other studies demonstrate that both R2 and R3α are necessary to target PP4c to the centrosomes^16^. Two studies suggest that the R3 subunit can target PP4c to its substrate. Human R3α, SMEK1, is reported to interact with the cell polarity protein Par3 to mediate its dephosphorylation by PP4c in the regulation of neuronal differentiation^17^. Furthermore, yeast R3, Psy2, specifically interacts with the glucose signal transducer protein Mth1 as a prerequisite for the PP4c–R3-mediated dephosphorylation of both Mth1 and a transcriptional repressor, Rgt1 (see ref. 18). These studies suggest that R3s are substrate-targeting subunits of PP4 but how substrate specificity is achieved remains unknown.

R3 orthologues are conserved throughout evolution with similar domain architecture and have been found from yeasts to human^11^,^17^,^18^,^19^ (Fig. 1a). They possess a predicted, conserved pleckstrin homology (PH) superfamily-like domain and Smk-1/DUF625 (Domain with Unknown Function 625, which is present in Smk-1 protein, a component of the IIs longevity pathway that regulates aging in *Caenorhabditis elegans*^20^) domain occupying the amino-terminal region of the protein. These domains are followed by a variable number of ARM (armadillo/HEAT repeats) in the middle and a carboxy-terminal unstructured (low complexity region) tail, which varies in length between different species (Fig. 1a).

Flfl^14^, the R3-type subunit of Drosophila PP4, has been shown to bind Miranda (Mira) and so recruits PP4 (PP4c–R2–R3^Falafel^) to regulate asymmetric division of neuroblasts^19^. However, PP4’s substrate in this process is unknown. Here we show that Flfl directly binds the centromeric protein C (CENP-C) and brings PP4 activity to centromeres. CENP-C is the key centromeric protein that provides a platform for kinetochore assembly and so bridges the mitotic centromere with core kinetochore proteins, which is critical for proper chromosome segregation^21^,^22^. We have precisely dissected the binding surfaces between Flfl and CENP-C and now present the first crystal structure of the EVH1 domain (which belongs to the family of PH-like domains^23^) of the R3 subunit of PP4 in complex with CENP-C. Interestingly, the sequence defining this new variant of the EVH1 fold and the residues crucial for CENP-C/substrate binding are well conserved in all orthologues of PP4R3. We show that PP4 activity is required for the dephosphorylation of both Flfl and CENP-C. Moreover, the Flfl–CENP-C interaction brings PP4 catalytic activity to centromeres and this is critical in regulating the integrity of the mitotic centromeres and associated kinetochore proteins. Thus these functional and structural data provide new insights into PP4 function and the regulation of cell division.

## Results

### PP4 directly interacts with CENP-C

We have previously demonstrated that Drosophila PP4 has critical roles in cell cycle progression^8^. As with other members of the PP2A family, the specificity of PP4 is thought to lie with interactions governed by its regulatory subunits. Therefore we wished to identify proteins interacting with Flfl, the R3-type subunit of Drosophila PP4. To this end, we established a Drosophila D.Mel-2 cell line expressing a Flfl::protein A fusion protein that we could affinity purify in complex with its associated proteins. Mass spectrometry identified components of the PP4 trimer consisting of Flfl (bait), PP4c and R2. CENP-C was also identified with high coverage and Mascot scores, indicating that it is a prominent partner of Flfl in this cell line (Table 1). To confirm that CENP-C also interacts with Flfl, we carried out the reciprocal experiment of establishing a D.Mel-2 cell line expressing a CENP-C::protein A. In this case, mass spectrometry identified all three subunits of PP4, including Flfl (Table 2). Moreover, similar results were obtained using either protein A-tagged Flfl or CENP-C expressed in Drosophila syncytial embryos (Supplementary Tables 1 and 2). Thus the interaction is not unique to cultured cells and is present during normal Drosophila development.

**Figure 1:**
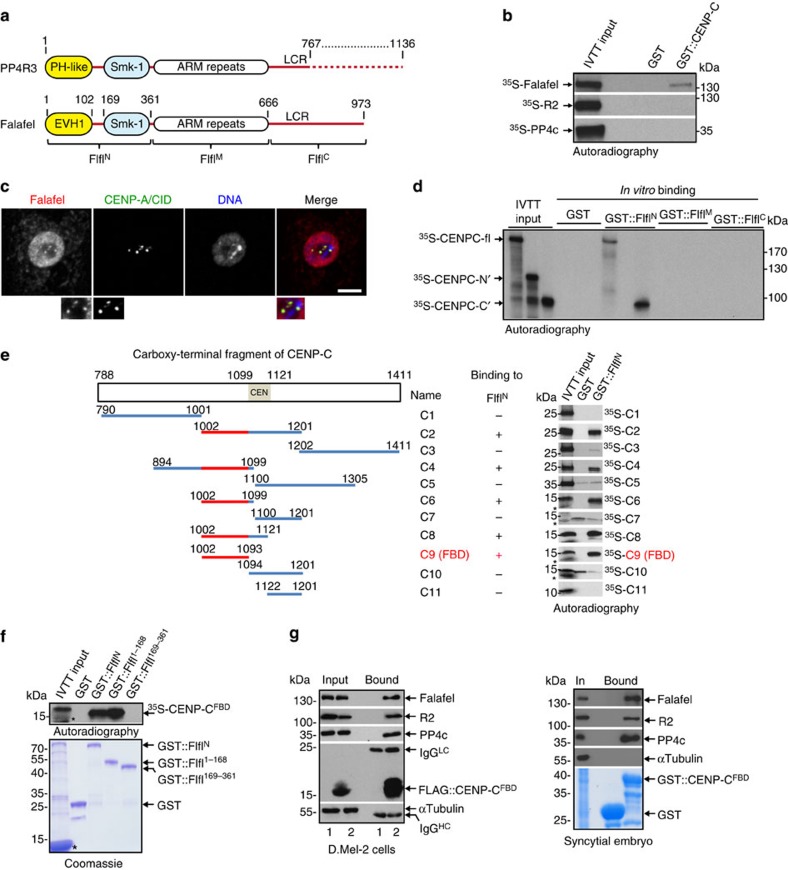
Interacting domains of Falafel and CENP-C. (**a**) Schematic representation of the common structural elements of PP4R3 proteins compared with *Drosophila* R3, Falafel. R3s contain a conserved PH/EVH1-like domain and Smk-1 domain with unknown function in their N-termini, followed by Armadillo/HEAT repeats (ARM) in the middle and a variable length (dotted line, according to HomoloGene R3 orthologues can be 767 to 1,136 amino acid-long) low complexity region (LCR) at the C-termini. Falafel fragments used in this study are indicated below. (**b**) *In vitro* binding of GST-tagged CENP-C with IVTT-expressed ^35^S-Met-labelled Falafel but not with labelled R2 or PP4c. (**c**) Confocal microscopy of interphase D.Mel-2 cells showing nuclear and centromeric enrichment of Falafel. Magnified images below show co-localization of Falafel with the centromeric protein CENP-A/CID. Scale bar, 5 μm. (**d**) *In vitro* binding of GST-tagged N-terminal (Flfl^N^, see **a**), (but not Middle and C-terminal Flfl^M^ and Flfl^C^, see **a**) fragment of Falafel with ^35^S-Met-labelled full-length (fl) and C-terminal part (C′) but not with N-terminal part (N′) of CENP-C. (**e**) Identification of the Falafel Binding Domain (FBD/fragment C9), a 92-amino acid-long region of the C-terminal part of CENP-C that can bind Flfl^N^ *in vitro*. CEN indicates the centromeric localization motif^58^. ‘*’ indicates globin from the reticulocyte lysate. (**f**) The EVH1 domain-containing GST-tagged Flfl^N^ (aa 1–361) and its truncated form Flfl^1–168^ (aa 1–168) specifically bind to ^35^S-Met-labelled CENP-C^FBD^ *in vitro*. In contrast Flfl^169–361^ (aa 169–361), the EVH1-lacking part of Flfl^N^, which includes only an Smk-1 domain, cannot interact with ^35^S-CENP-C^FBD^. ‘*’ indicates globin from the reticulocyte lysate. (**g**) Western blots revealing that the entire PP4 complex is co-precipitated with FLAG::CENP-C^FBD^ from cultured cells or syncytial embryos; anti-αTubulin provides a loading and negative control. Left hand panels show Western blots of cultured cells expressing FLAG only (1) or FLAG::CENP-C^FBD^ (2). Right hand panels show Western blots of syncytial embryo proteins purified on immobilized GST::CENP-C^FBD^. GST only serves as a negative control. Coomassie-stained gels demonstrate the loading of the bait proteins.

To determine which of the three PP4 subunits, if any, directly interacts with CENP-C, we synthesized each ^35^S-Met-labelled subunit using *in vitro* transcription–translation (IVTT) and identified which would bind to immobilized glutatione S-transferase (GST)-tagged full-length CENP-C. Of the three subunits, only Flfl directly interacted with CENP-C (Fig. 1b).

As a major centromeric component, CENP-C shows distinct centromeric localization throughout the cell cycle. To determine whether Flfl also localizes at centromeres, we generated an anti-Flfl antibody (Supplementary Fig. 1a) and used this to investigate Flfl’s sub-cellular localization by indirect immunofluorescence (IF). This revealed Flfl to be a predominantly nuclear protein, a proportion of which co-localized with a centromeric marker, the histone variant CENP-A (centromere identifier (CID), in Drosophila; Fig. 1c). This accords with the association of Flfl with centromeric CENP-C as shown by proteomics and *in vitro* binding assays. Together these data indicate that PP4 is a component of the Drosophila centromere that associates with CENP-C through a direct interaction made by its R3 subunit, Flfl.

### Flfl binding domain of CENP-C recruits PP4 to centromeres

To narrow down the interacting surfaces between Flfl and CENP-C, we employed an *in vitro* binding assay and found that the carboxy-terminal part of CENP-C specifically binds the amino-terminal part of Flfl (Fig. 1d, Supplementary Fig. 1b), which contains an EVH1 domain, a member of the PH-like superfamily (residues 1–102) and an Smk-1 domain (residues 169–361) separated by a short inter-domain region (residues 103–168; Fig. 1a). Subsequently, we determined that a 92-amino acid (aa)-long fragment within CENP-C (the Flfl Binding Domain (FBD); CENP-C residues 1002–1093) and the first 168 aa of Flfl (Flfl^1–168^) were sufficient to support this interaction (Fig. 1e,f). Finally, by using recombinant CENP-C^FBD^ and Flfl^1–168^ expressed in bacteria and purified to homogeneity, we demonstrated that the two protein fragments can form a stable stoichiometric complex *in vitro* confirming the direct and suggesting a phosphorylation-independent interaction between them (Supplementary Fig. 1c).

Just as we could use full-length CENP-C expressed in D.Mel-2 cells as an affinity bait to purify the PP4 holoenzyme (Table 2), we could achieve the same purification using FLAG::CENP-C^FBD^ (Fig. 1g). Moreover, all three subunits of PP4 could be successfully co-purified from Drosophila syncytial embryo extracts, when recombinant GST::CENP-C^FBD^ protein was used as a bait (Fig. 1g), suggesting that CENP-C^FBD^ is sufficient for the binding of the PP4 holoenzyme. In accord with these findings, CENP-C co-purified with protein A::Flfl^1–168^ from cultured cells (Supplementary Table 3), but not with protein A::Flfl^169–973^ (Supplementary Table 4). Using indirect IF we found that, similar to the endogenous protein (Fig. 1c), both FLAG-tagged Flfl and Flfl^1–168^ localize to centromeres throughout the cell cycle (Supplementary Fig. 1d). By contrast, FLAG::Flfl^169–973^ was completely excluded from centromeres (data not shown). This strongly suggests that the EVH1 domain within Flfl^1–168^ is both necessary and sufficient to target Flfl to CENP-C and so to centromeres.

### Flfl-interacting motif of CENP-C is an atypical EVH1 ligand

**Figure 2:**
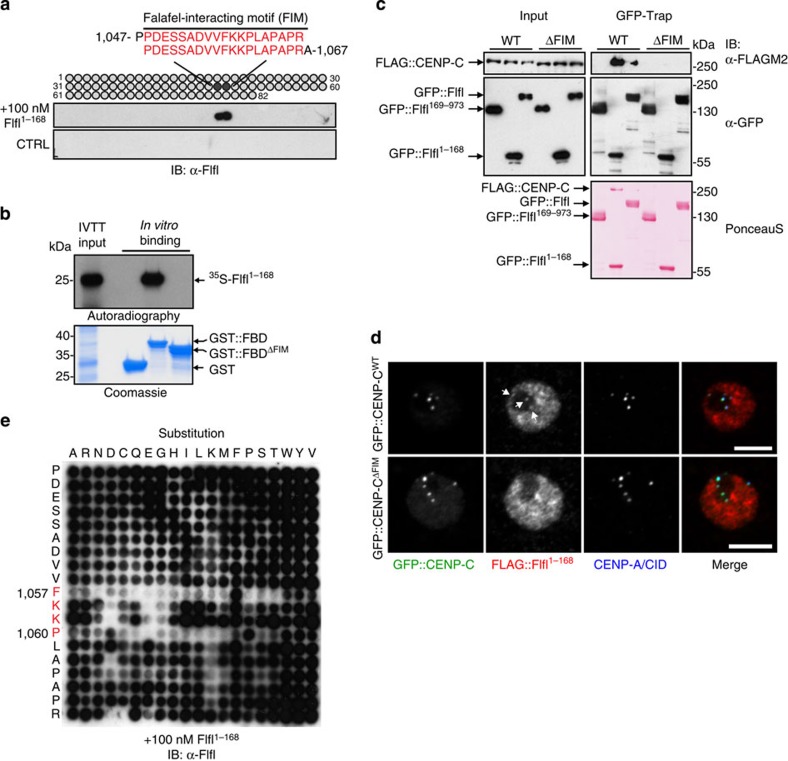
CENP-C′s FIM motif is crucial for Falafel binding and recruitment to the centromere. (**a**) Peptide array: Flfl^1–168^ binds to two 20-mer peptides in CENP-C^FBD^ identifying the 19-amino acid-long Falafel-interacting motif (FIM). (**b**) ^35^S-Met-labelled Flfl^1–168^ is able to show *in vitro* binding to GST-tagged wild-type FBD (GST::FBD) but not to the FIM-deleted FBD (GST::FBD^ΔFIM^). (**c**) GFP–Trap purification of full-length Falafel (GFP::Flfl) or its 1–168 amino acid fragment (GFP::Flf^1-168^) co-precipitates FLAG-tagged CENP-C (FLAG::CENP-C). This interaction is lost following deletion of the FIM (ΔFIM). GFP::Flfl^169–973^ provides a negative control. Ponceau S staining assesses loading. (**d**) D.Mel-2 cells depleted of endogenous CENP-C and co-expressing FLAG::Flfl^1–168^ with either GFP::CENP-C^WT^ or GFP::CENP-C^ΔFIM^. In the absence of FIM, Flfl^1–168^ no longer localizes to the centromere. Arrows indicate centromeric FLAG::Flfl^1–168^ signals. Scale bars, 5 μm. See also Supplementary Fig. 1e. (**e**) Peptide substitution array indicating that the sequence ^1057^Phe–Lys–Lys–Pro^1060^ within CENP-C is critical for Flfl^1–168^ binding *in vitro*.

We asked whether the FBD of CENP-C might have features in common with known EVH1 ligands that contain high and repetitive proline content^24^. Therefore to more precisely define the interaction site, we probed an array of overlapping 20 aa peptides covering the entire CENP-C^FBD^ fragment with recombinant Flfl^1–168^. This identified a 19 aa sequence located in the middle of the FBD (CENP-C residues 1048–1066), which strongly interacted with Flfl^1–168^ that we termed Flfl-interacting motif (FIM; Fig. 2a). We then tested the functional importance of this sequence by carrying out *in vitro* binding assays, which showed that the deletion of the FIM from CENP-C^FBD^ prevented its interaction with ^35^S-Met-labelled Flfl^1–168^ (Fig. 2b). Although both the FIM deletion mutant of full-length CENP-C (FLAG::CENP-C^ΔFIM^) and the wild-type protein (FLAG::CENP-C^WT^) localized to centromeres equally well, the FLAG::CENP-C^ΔFIM^ protein was unable to interact with full-length Flfl or Flfl^1–168^ in co-immunoprecipitation experiments (Fig. 2c) or with any of the PP4 subunits as indicated by proteomics (Supplementary Tables 5 and 6). Thus the FIM appeared to be indispensable for the complex formation within the context of full-length CENP-C protein. Finally we verified the role of FIM in the recruitment of Flfl–PP4 to centromeres. We found that FLAG::Flfl^1–168^ localized to centromeres when endogenous CENP-C was replaced with green fluorescent protein::CENP-C^WT^ (GFP::CENP-C^WT^), but not at all when replaced with GFP::CENP-C^ΔFIM^ (Fig. 2d and Supplementary Fig. 1e). Thus a 19-aa-long segment of CENP-C, the FIM, is crucial for the recruitment of Flfl and hence PP4 to centromeres.

As the FIM did not show any particular enrichment for proline residues characteristic of EVH1 ligands, we decided to define individual residues within this sequence that were important for the interaction. We found that substitution mutants at four adjacent sites within the 19-mer, 1057-Phe-Lys-Lys-Pro-1060 (FKKP), disrupted this binding suggesting their potential importance in making direct contact between the two proteins (Fig. 2e). The phenylalanine and proline residues appear absolutely critical for this interaction, while some variation in the identity of the intervening residues may be tolerated.

### Conserved structure of the EVH1 domain of Flfl

**Figure 3:**
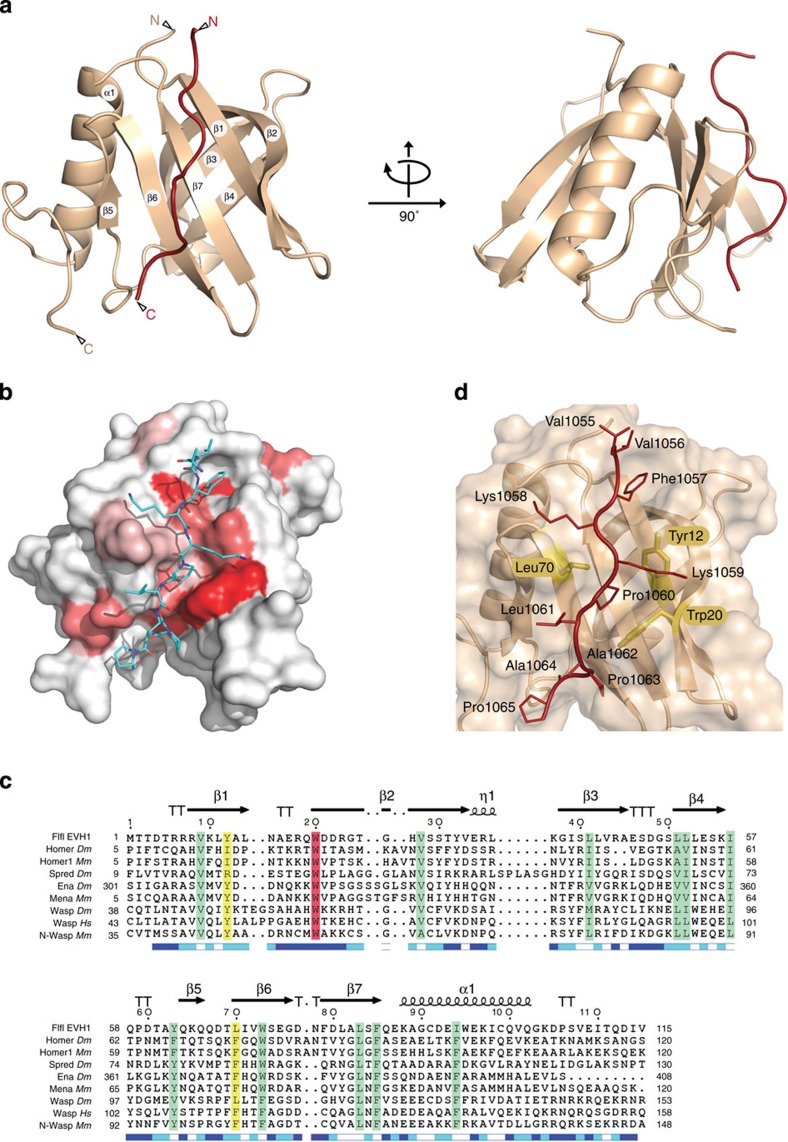
Structure of the Flfl^1–122^–CENP-C^FIM^ complex. (**a**) Ribbon diagram of the Falafel Pleckstrin Homology-like domain (Flfl^1–122^; wheat) with CENP-C FIM peptide (red) in two different orientations. (**b**) Representation of Flfl^1–122^ surface conservation coloured from red (highly conserved) to white (non-conserved). The deepest red ‘shelf’ lying in the middle of Flfl^1–122^ is due to the highly conserved Trp20. (**c**) Sequence alignment of EVH1 domains from Homer, Spred, Ena, Mena, Wasp and N-Wasp. Species are *Mm* (Mouse), *Hs* (Human) and *Dm* (*Drosophila*). Secondary structural elements of the Flfl^1–122^ are represented by arrows (β strands), squiggles (α helices) and T (turns). Highly conserved residues are coloured in green. The invariant tryptophan is coloured in red and residues involved in proline stacking in EVH1 but not Flfl^1–122^ in yellow. The relative accessibility of each residue is rendered as blue-coloured boxes located at the last line of each block. The blue scale is set as follows: blue, accessible; cyan, intermediate and white, buried. The Flfl^1–122^ deviates from the canonical EVH1 domain; a conserved phenylalanine (Phe77 in 1EVH) is replaced by leucine (Leu70) and the side chain of a conserved tyrosine (Tyr12 in Flfl^1–122^) is in a different orientation to that in 1EVH and other EVH1 family members. (**d**) Close up view of Flfl^1–122^–FIM interaction site. CENP-C residues have been labelled and three critical residues from the Flfl^1–122^ ligand recognition site are highlighted in yellow. Phe1057 occupies a hydrophobic pocket composed largely of the methylene groups of a series of hydrophilic amino acid side chains, while Pro1060 of CENP-C is sandwiched between Leu70 and the highly conserved Trp20.

To better understand the molecular basis for the Flfl–CENP-C interaction, we co-crystallized an amino-terminal 122-aa-long fragment of Flfl containing the EVH1 domain (Flfl^1–122^) and the CENP-C^FIM^ peptide and solved the structure by molecular replacement to 1.5 Å resolution as described in Methods. Crystallographic statistics and representative electron density are provided in Table 3 and Supplementary Fig. 2a, respectively. The final model comprises residues 4–114 of Flfl and 1055–1065 of CENP-C. We found that the Flfl^1–122^ fragment is composed of a seven-stranded β barrel capped by a C-terminal α-helix (Fig. 3a). Two concave surfaces are formed by the outside of each β sheet, one of which (strands β1, β2, β5, β6 and β7; Fig. 3a) forms the peptide-binding groove with contributions from an inter-strand loop (residues 66–69). The CENP-C peptide can be unambiguously identified as bound into that groove (Supplementary Fig. 2b). Although the structure of Flfl^1–122^ slightly deviates from the canonical EVH1 domains (Supplementary Fig. 2c), three-dimensional searches using the DALI server^25^ showed several proteins in the EVH1 family^23^ as close structural homologues. The EVH1 domain from MENA^26^ appears most closely related structurally (root-mean-square deviation 2.2 Å over 113 Cα positions; *Z*-score=13.8). The binding site lies in a highly conserved groove on the surface of the protein (Fig. 3b,c) burying 1,700 Å^2^ of the total 6,700 Å^2^ surface of Flfl^1–122^ and corresponds to the surface responsible for binding proline-rich sequences in other members of the EVH1 family^27^. The residues within the EVH1 domain, which are involved in binding to CENP-C^FIM^, are highly conserved in the R3 subunits of PP4 from a very wide range of other organisms (Supplementary Fig. 2d).

Residues 1055–1065 of the CENP-C^FIM^ peptide are well defined in the electron density, and include FKKP motif of the residues predicted to interact with the Flfl^1–122^ by peptide array analysis (Fig. 2e). The peptide backbone adopts a left-handed helical conformation similar to the polyproline II (PPII) helix^28^. This arrangement is mainly stabilized by the establishment of hydrogen bonding and van der Waals interaction between Pro1060 and Trp20. The twofold pseudo-symmetry displayed by the PPII helix enables bidirectional ligand binding often observed in polyproline recognition motifs such as the SH3 domain^29^,^30^. Additional residues flanking the PPII sequence provide directional specificity. In our structure CENP-C′s Phe1057 and Pro1060 provide the principle specificity and directionality of binding (Fig. 3d and Supplementary Fig. 2e; see also the legend for Supplementary Fig. 2e). EVH1 domain ligand selectivity is dependent on which family the domain belongs to^27^. Class I EVH1 domains, as found in the Ena/VASP proteins recognize FPPPP sequences, while Class II domains, exemplified by Homer/Vesl, bind a PPxxF motif. Class III domains, found uniquely in WASP proteins are specific for LPPPEP. The ligands for Class IV domains, also known as Spred domains, are unknown. A structural study has suggested that the Spred-1 EVH1 domain may preferentially bind low proline content ligands due to a narrowed peptide-binding channel with slight displacement of the conserved tryptophan and associated strand β2 (ref. 31). However, in our structure this element more closely resembles the canonical EVH1 fold. Although the FKKP motif recognized by Flfl closely resembles the inverted PPxxF sequence bound by Homer, the mGluR-Homer crystal structure shows an unusual peptide conformation, in which the peptide backbone arches away from the EVH1 surface to place the terminal phenylalanine in a unique binding pocket^32^. In our structure, the FIM is bound in the reverse orientation to that of the mGluR peptide, placing the phenylalanine in a roughly similar location. However, the peptide is bound to Flfl in a more classical configuration that resembles Class I and Class II ligands by maintaining PPII configuration for its full length. We suggest that Flfl–FIM interaction constitutes a novel mode of EVH1 binding in which a low proline content ligand is recognized in a PPII conformation.

### PP4 is necessary for the integrity of mitotic centromere

**Figure 4:**
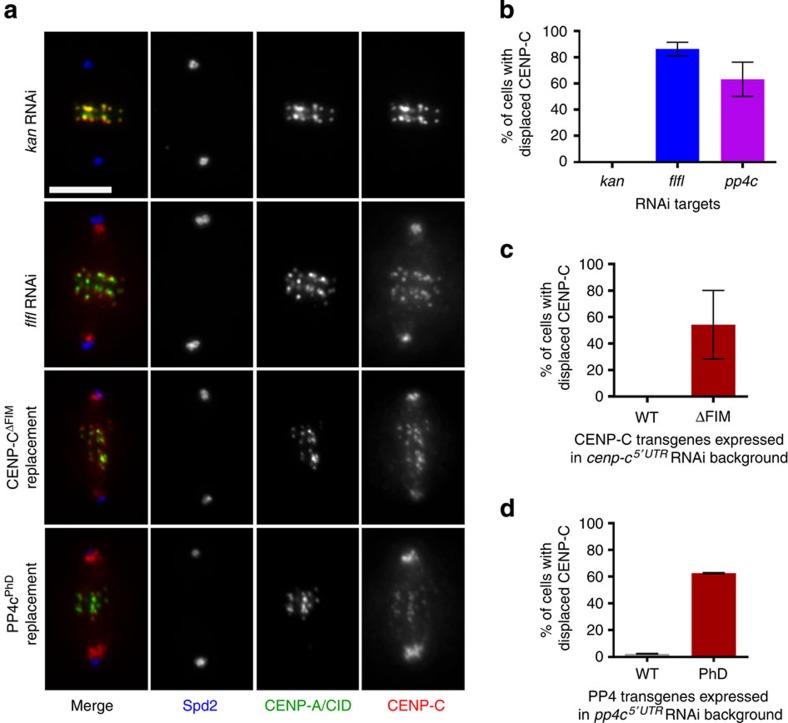
Presence and catalytic activity of PP4 are required to regulate mitotic centromere integrity. (**a**) A proportion of CENP-C is mislocalized from the centromeres and accumulated along the spindle and spindle poles in *flfl*-depleted mitotic cells. *kan* RNAi is used as a negative control. The same phenotype is observed after replacement of endogenous CENP-C with FLAG::CENP-C^ΔFIM^ or replacement of endogenous PP4c with FLAG::PP4^PhD^. Scale bar, 5 μm. Spd2, centrosome marker; CENP-A/CID, centromere marker. (**b**) Proportion of mitotic cells showing CENP-C mislocalization on *kan* (control), *flfl* or *pp4c* depletions. (**c**) FLAG::CENP-C^ΔFIM^ cannot complement centromeric function of depleted endogenous CENP-C. (**d**) FLAG::PP4c^WT^ can fully rescue CENP-C displacement resulting from depletion of endogenous PP4c, whereas the phosphatase-dead form (FLAG::PP4c^PhD^) cannot. Bars represent s.d. in all three graphs; *n*>50 for each condition.

Having defined the interaction site between Flfl and CENP-C, we then wished to assess its biological significance. To this end we first depleted cells of Flfl and recorded the behaviour of centromeres throughout the cell cycle. We did not observe any consequences of Flfl depletion in interphase cells. However, during mitosis a proportion of CENP-C became displaced from centromeres and accumulated on the spindle microtubules or around the spindle poles. In contrast, CENP-C was entirely restricted to centromeres in control cells (*kan* RNAi versus *flfl* RNAi; top two panels in Fig. 4a; quantitation in Fig. 4b). CENP-C serves a fundamental role at the centromere both as a component of the centromere itself and as a partner of the kinetochore-associated Mis12 complex^21^,^22^. We therefore investigated whether CENP-C′s centromeric displacement also affected the mitotic localization of any of the core kinetochore proteins. We found that while the centromeric protein, CENP-A, remained associated with the centromere, the displaced CENP-C was accompanied by all core kinetochore components, exemplified by Mis12, Nsl1 and Spc105 (Supplementary Fig. 3).

To address whether the direct binding of Flfl to CENP-C was responsible for maintaining centromere integrity during mitosis, we determined the consequences of specifically disrupting the CENP-C–Flfl interaction. To this end we substituted endogenous CENP-C with FLAG-tagged CENP-C deleted for the FIM (FLAG::CENP-C^ΔFIM^). This not only led to the removal of Flfl from interphase centromeres, but also resulted in the displacement of CENP-C^ΔFIM^ from centromeres in mitosis (Fig. 4a,c). Thus when CENP-C fails to recruit Flfl, the integrity of the mitotic centromere is compromised in a manner similar to that occurring following Flfl depletion.

**Figure 5:**
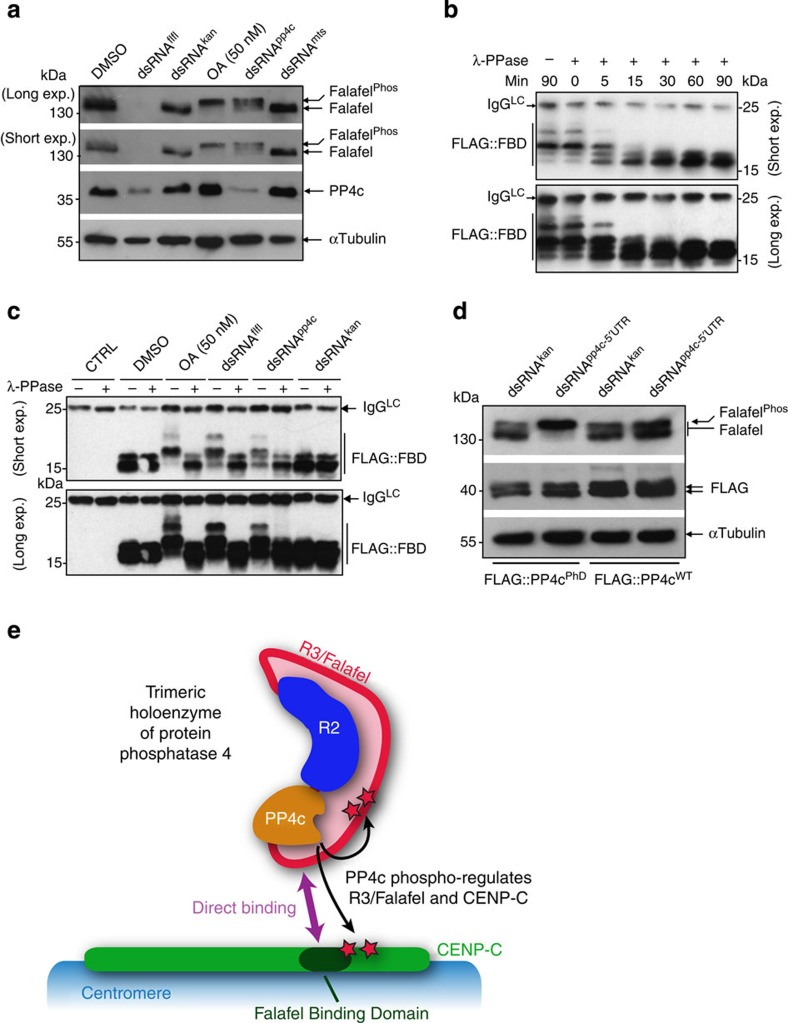
PP4 phosphoregulates Falafel and CENP-C. (**a**) Treatment of D.Mel-2 cells with interfering ds *flfl* RNA leads to reduction of both Falafel and PP4c, suggesting that the depletion of a regulatory subunit of PP4 results in the destabilization of the catalytic subunit (as observed also for PP2A (ref. 8)). Falafel migrates as a doublet. Treatment with 50 nM okadaic acid (OA) indicates that the upper band represents a phosphorylated form. On depletion of PP4c (dsRNA^*pp4c*^) but not PP2Ac (dsRNA^*mts*^) the phosphorylated form of Falafel predominates, suggesting that Falafel is a novel PP4 substrate. αTubulin is loading control. (**b**) The CENP-C^FBD^ fragment is phosphorylated *in vivo*. FLAG::FBD undergoes shifts in its electrophoretic mobility following treatment *in vitro* with λ-PPase. Samples were run on Phos-tag SDS–PAGE followed by immunoblotting. (**c**) Phospho status of CENP-C^FBD^ depends on the presence of PP4. Extracts of cells expressing FLAG::FBD treated with nothing (control), DMSO, 50 nM okadaic acid (OA) and interfering dsRNAs targeted against *flfl* (dsRNA^*flfl*^), *pp4c* (dsRNA^*pp4c*^) and control (dsRNA^*kan*^). Phosphorylated forms are seen following okadaic acid treatment and following depletion of Falafel or PP4c, indicating that the PP4 holoenzyme is required to maintain the dephosphorylation status of FLAG::FBD *in vivo*. (**d**) Electrophoretic mobility of Falafel in extracts of cells after control RNAi (dsRNA^*kan*^) or RNAi directed against the 5′ untranslated region (UTR) of *pp4c* (dsRNA^*pp4c-5*′*UTR*^). Cells are expressing either the transgenic FLAG-tagged phosphatase-dead variant of PP4c (FLAG::PP4c^PhD^) or its wild-type counterpart (FLAG::PP4c^WT^). Note that Falafel migrates as its phosphorylated form in the FLAG::PP4c^PhD^-expressing cell line, indicating that the PP4 catalytic activity is required for Falafel dephosphorylation *in vivo*. (**e**) A hypothetic model demonstrating that PP4 activity is localized at centromeres in *Drosophila* cells. Regulatory subunit 3, Falafel, directly interacts with the Falafel Binding Domain of CENP-C (violet arrow), which brings the trimeric holoenzyme of PP4 to centromeres. Centromeric PP4 activity is important for the integrity of mitotic centromeres and affects the phospho status of Falafel and CENP-C (highlighted as red stars).

To address whether the mitotic centromere association of CENP-C requires PP4’s catalytic activity, we first depleted its catalytic subunit PP4c. This resulted in partial CENP-C displacement from centromeres in mitotic cells (Fig. 4b), similar to the effects of Flfl depletion. However, we could not rule out the possibility that the CENP-C mislocalization phenotype represented loss of a structural function for the PP4c subunit and so we decided to develop a catalytically inactive form of this phosphatase. To do this we first needed to identify changes in the phospho-modifications of proteins associated with loss of PP4. Interestingly, we noticed that on PP4c depletion, Flfl remained hyper phosphorylated and migrated slower in a band-shift assay (Fig. 5a). We also found that the FLAG-tagged FBD of CENP-C displayed lambda phosphatase (λ-PPase) sensitive bands (Fig. 5b) of higher electrophoretic mobility after depletion of either Flfl, PP4c or in the presence of phosphatase inhibitor, okadaic acid (Fig. 5c). These observations suggest that both Flfl and CENP-C are novel substrates of PP4, although we cannot rule out the possibility of the indirect inhibition of some secondary phosphatase on PP4 knockdown. In addition, these findings provided us with a simple assay for assessing the catalytic activity of PP4c. To design a catalytically inactive PP4c, we first aligned the primary sequences of the catalytic subunits of PP2Ac/mts and PP4c, which identified highly conserved residues in the active site regions^33^ (Supplementary Fig. 4a). We then engineered a transgene with the aa substitutions, D85N and H115N, which should render the phosphatase catalytically inactive. Consistent with the loss of enzymatic activity, D.Mel-2 cells expressing this phosphatase-dead (PhD) mutant of PP4c in the absence of its endogenous counterpart no longer displayed a Flfl doublet on immunoblots (IBs) but only the slower migrating, phosphorylated form of the protein (Fig. 5d). This phosphatase-dead counterpart of PP4c (PP4c^PhD^) could interact with both R2 and Flfl (Supplementary Fig. 4b), confirming the structural integrity of the inactive trimeric phosphatase. We then expressed PP4c^WT^ or PP4c^PhD^ in D.Mel-2 cells lacking endogenous PP4c (Supplementary Fig. 4c) and assessed their progression through mitosis. The loss of PP4 catalytic activity resulted in CENP-C displacement from the centromeres (Fig. 4a, bottom panels) to virtually the same extent as both Flfl and PP4c depletions (compare Fig. 4d with Fig. 4b and c). Thus, centromere integrity during mitosis requires the localized delivery of the catalytic subunit to the centromere mediated by the interactions of PP4’s R3 regulatory subunit, Flfl and the centromeric protein CENP-C.

## Discussion

Studies of protein phosphatases have been limited by restricted knowledge of either the mechanisms that regulate their specificity or the identity of the protein kinases they oppose. Moreover, the fact that most protein phosphatases are multimeric complexes, in which regulatory subunits deliver stability, localization and substrate recognition activities to the catalytic subunits, has limited the number of inhibitory compounds that have been developed against them^1^,^34^,^35^. Together, this has led to a significant delay in our understanding of how exactly phosphorylation regulates multiple cellular processes.

We have developed an interest in PP4 as a newly emerged regulator of cell cycle progression^8^,^9^. PP4 is a member of the PP2A family of Ser/Thr phosphoprotein phosphatases and as in the case of PP2A, the common form of the holoenzyme comprises a catalytic and two regulatory subunits. We were specifically led to study potential roles of Flfl, the R3 subunit of PP4 in Drosophila, because of the involvement of R3 subunits in a variety of cellular processes^3^,^11^ including the cell cycle^16^. The human R3β subunit SMEK2, for example, participates in regulation of S-phase progression and behaves as a chromatin protein phosphorylated by the cyclin-dependent kinases^36^. In addition, R3 subunits of PP4 influence cell cycle progression through involvement in DNA repair pathways^6^,^7^. Our present finding of a functional interaction between Flfl and CENP-C directly illustrates the role played by the R3 subunit in targeting the PP4 to the centromere to regulate its structure and function.

Several previous studies have implicated R3 as the targeting subunit of PP4 (see introduction). This regulatory subunit is well conserved from yeast to human and has an EVH1 domain and a Smk-1/DUF625 domain occupying the N-terminal part of the protein (Fig. 1a). This part of the molecule appears important in all of its targeting interactions described to date: the Smk-1/DUF625 domain of mammalian R3α interacts with the PP4 substrate Par3 in neuronal differentiation^17^; the EVH1 domain of yeast R3 interacts with the PP4 substrate Mth1 (see ref. 18) and the EVH1 (formerly RanBD) domain of Drosophila R3, Flfl, binds Mira to regulate asymmetric division of neuroblasts^19^. We now show that the EVH1 domain of Flfl also directly interacts with the key centromeric protein, CENP-C.

EVH1 domains are structurally related to PH domains but generally bind to proline-rich amino-acid sequences rather than phospholipids. Our study provides the first analysis of the interaction between an R3 regulatory subunit and its target at atomic resolution that together with *in vitro* and *in vivo* binding assays unequivocally confirms complex formation between these proteins. This mode of targeting PP4 to its substrate(s) appears very different to other Ser/Thr protein phosphatases such as PP1 and PP2A, in which the variable regulatory subunits often form a contiguous substrate recognition surface with the conserved catalytic subunit^37^. The R3 regulatory subunits of PP4 by contrast appear to rely on a flexibly linked EVH1 domain for substrate recruitment, which then presumably places the catalytic subunit proximal to the target residue(s). Analysis of the crystal structure shows that the interaction takes place in the groove that forms within the EVH1-like domain of Flfl. This domain deviates from the canonical EVH1 domain in that a conserved phenylalanine (Phe77 in 1EVH) is replaced by leucine (Leu70) and the side chain of a conserved tyrosine (Tyr12 in Flfl^1–122^) is in a different orientation to that in other EVH1 family members (Fig. 3c and Supplementary Fig. 2c). Nevertheless, the 3D organization of this part of Flfl shows close structural homology with other EVH1 domains. Moreover, EVH1 domains present in amino termini of R3 subunits of PP4 phosphatases are highly conserved at their primary sequence, suggesting that their mode of binding to ligands will also be conserved (Supplementary Fig. 2d). Interestingly, unlike other EVH1 ligands, the segment of CENP-C that binds Flfl does not have an enriched and repetitive proline content. Within the 19 aa FIM of CENP-C, we identify a stretch of four aa of which Phe1057 and Pro1060 are key for specificity of binding. They also direct the orientation of binding within the EVH1 groove. This evokes a direct comparison with the crystal structure of the Homer EVH1 domain bound to an mGluR peptide that contains the reverse PxxF recognition motif^32^. It seems, however, that the Homer EVH1 domain might be more of an exception to the rule since the FxxP sequence in CENP-C appears to adopt a conformation extremely similar to the left-handed PPII helix, which more typically interacts with the EVH1 domains. It is of future interest to examine the variations allowed at this interface in considering the targeting of PP4 to its multiple sites of action via its R3 subunit, and how the remainder of the R3 domain may participate in substrate binding.

Our results point to the functional importance of the interaction between the Flfl EVH1 domain and CENP-C in bringing the catalytic subunit PP4c to centromeres. Failure to do this results in some loss of integrity of the centromere during mitosis that we demonstrate in several ways. First, cells depleted of Flfl exhibit displacement of CENP-C away from centromeres and towards the centrosome during mitosis, whereas localization of the centromeric histone CENP-A/CID remains unaffected. This specifically requires the interaction between Flfl and CENP-C because the same phenotype is evoked when endogenous CENP-C is substituted by a CENP-C^ΔFIM^ mutant, which does not bind Flfl. Two pieces of evidence suggest that Flfl’s function in this context is to deliver protein dephosphorylation activity to centromeres: CENP-C also becomes displaced from centromeres during mitosis either following depletion of the PP4 catalytic subunit, PP4c, or when Phosphatase Dead PP4c is substituted for the endogenous wild-type protein.

CENP-C is not only a structural component of the centromere but it also provides a scaffold for kinetochore assembly and a hub for kinetochore regulation^21^,^22^,^38^. We have previously observed that mislocalization of CENP-C at the centrosome through other means results in mislocalization of kinetochore proteins^21^, just as we now observe following loss of PP4 activity from the centromere. The extensive phospho-modification of CENP-C represents a tremendous technical challenge to the unravelling of the precise patterns of protein phosphorylation and dephosphorylation that regulate its function. Genome-wide phospho-proteomics studies have previously found CENP-C to be heavily phosphorylated at multiple sites^39^,^40^, suggesting that it may be phosphorylated by multiple protein kinases. Our own analysis identifies at least 20 sites on CENP-C phosphorylated *in vivo* (David Glover lab, unpublished data). Although the direct roles of protein phosphatases in dephosphorylating these sites must first await the identification of the opposing protein kinases, it seems very likely that both multiple kinases and multiple phosphatases will be involved.

CENP-C together with CENP-A/CID and CAL1 forms a complex at the Drosophila centromere, and the three proteins show interdependency in their localization^41^. Our observations imply that in the absence of the centromeric function of PP4, the association of CENP-C with its other centromeric partners is weakened, either during loading or maintenance at centromeres, allowing its displacement towards spindle poles. While our findings implicate CENP-C as a putative PP4 substrate, it seems reasonable to assume that it is not the only centromere/kinetochore protein dephosphorylated by PP4. Indeed it has been described, for example, that in human cells PP4 phosphoregulates Ndel1, the human orthologue of Drosophila NudE^42^, which is critical for the recruitment of dynein to kinetochores^43^ and also regulates microtubule organization^42^,^44^. Another study finds the kinetochore component Dsn1 as a potential target of PP4 (see ref. 5). Thus dephosphorylation of alternative kinetochore substrates could also influence centromeric protein function.

The central finding of our study is that a variant of an EVH1-like fold located in the amino-terminal part of Flfl, the R3 subunit of Drosophila PP4, is required to bind to a motif in the carboxy-terminal part of CENP-C and so to target PP4 phosphatase activity to regulate centromeric structure (Fig. 5e). This opens the door not only for studies on the structure and function of PP4 but also on which centromeric or kinetochore proteins might be regulated by PP4 activity and about their exact roles in kinetochore biology and the regulation of cell cycle progression.

## Methods

### DNA constructs

Complementary DNA clones for PP4c (RE58406; *CG32505*, *PP4–19c*), R2 (LD28993; *CG2890*; *PP4R2r*) and Flfl (LD13350; *CG9351, flfl/PP4R3*) were obtained from the Drosophila Genomics Resource Centre. Full length, N′ and C′ of CENP-C were made previously^21^. DNA encoding full-length CENP-C or its truncated forms (FBD (CENP-C^1002–1093aa^), FBD^ΔFIM^ (FBD^Δ47–65aa^) and CENP-C^1202–1411aa^ (C3)), full-length subunits of PP4 (PP4c, R2 and Flfl) and truncated forms of Flfl (Flfl^N^ (aa 1–361), Flfl^M^ (aa 362–666), Flfl^C^ (aa 667–973), Flfl^1–168aa^, Flfl^169–361aa^ or Flfl^169–973aa^) or PP4c (PP4c^1–50aa^) were respectively cloned into pDONR221 using the Gateway System (Life Technologies). CENP-C^Δ1048–1066aa^ (hereafter CENP-C^ΔFIM^) or PP4c^D85NH115N^ (hereafter PP4c^PhD^ as Phosphatase Dead) entry clones were created by standard mutagenesis using QuikChange II XL Site-Directed Mutagenesis Kit (Agilent Technologies). All entry clones were verified by DNA sequencing. Expression constructs were then made using the following destination vectors: pDEST15 (N-terminal GST fusion in *Escherichia coli*, Life Technologies), pDEST17 or pET-DEST42 (N- or C-terminal 6 × His fusion in *E. coli* or *in vitro* transcription/translation; Life Technologies), pAFW and pAWF (N- or C-terminal 3 × FLAG fusion in D.Mel-2 cells, Drosophila Gateway Vector Collection), pMT–PrA (N-terminal protein A fusion under the regulation of the copper-inducible *Metallothionein A* (*CG9470*) promoter in D.Mel-2 cells, in house), pMT–GFP (N-terminal eGFP-fusion in D.Mel-2 cells, in house) and pMAT–PrA–N/C (N- or C-terminal protein A fusion under the regulation of *maternal αtubulin* promoter in Drosophila embryos, in house). Conventional cloning was used to insert full-length CENP-C CDS into the pAc5.1_V5-ProtA vector (obtained from Paolo D’Avino, Department of Pathology, University of Cambridge) for constitutive expression with a protein A tag in D.Mel-2 cells^21^. His::TEV::Flfl^1–168aa^, His::TEV::Flfl^1–122aa^ (hereafter Flfl^1–122^) or His::TEV::CENP-C^FBD^ constructs were made by PCR amplification of the coding regions with a TEV protease cleavage site fused to their 5′ ends. The PCR products were inserted into the pETDuet-1 plasmid (Novagen) in frame with the N-terminal 6 × His-tag (MCS1). DNA constructs for the coupled IVTT expression of full-length untagged PP4c, R2 or Flfl were made by conventional cloning: CDS were amplified by PCR and inserted into the T7 promoter-regulated pETDuet-1 plasmid (MCS2). T7 promoter-driven pDEST17/CENPC-fl, CENPC-N′ or CENPC-C′ constructs used in IVTT were made by the LR reaction. Linear DNA fragments used in IVTT encoding truncated forms of CENP-C-C′ (C1–C11, see Fig. 1e) or Flfl (Flfl^1–168^) were generated by PCR using appropriate primers to create the following configuration: T7-Kozak-ATG-gene-specific sequence-STOP codon. Oligonucleotide primers used in this study are listed in Supplementary Table 7.

### Recombinant protein expression and purification

For crystallizations studies, His::TEV::Flfl^1–122^ was expressed in *E. coli* strain BL21 (DE3) RIL and purified to homogeneity as follows: cells were grown to A600=0.6 and expression was induced with 1 mM isopropyl 1-thio-β-D-galactopyranoside overnight at 18 °C. Cells were lysed by sonication in buffer containing 150 mM NaCl, 50 mM Tris–HCl, pH 8.0, 0.1 mM EDTA, 0.5 mM Tris(2-carboxyethyl)phosphine, complete protease inhibitor cocktail (Roche, 11873580001) and centrifuged at 34,000 *g* to pellet cell debris. The cleared lysate was loaded onto a 5 ml HisTrap HP column (GE Healthcare) and eluted with a gradient of 0–250 mM imidazole. His::TEV was removed by adding TEV protease (in house) to the main Flfl^1–122^-containing fractions, which then were further purified by anion-exchange chromatography using a 5 ml HiTrap Q Sepharose FF column (GE Healthcare) and eluted with a gradient of 0.15–1 M NaCl. The final purification was performed using size exclusion chromatography in buffer containing 150 mM NaCl, 50 mM Tris–HCl, pH 8.0, 0.5 mM Tris(2-carboxyethyl)phosphine. The purity of samples was analyzed by SDS–PAGE.

For other purposes, His- or GST-tagged proteins were expressed in Rosetta2(DE3)pLysS Singles (Novagen) *E. coli* strain as detailed above. GST-tagged proteins were affinity purified on Glutathione Sepharose 4b resin (GE Healthcare) according to the manufacturer’s protocol. GST-tagged Flfl^N^, Flfl^M^, Flfl^C^, Flfl^1–168^, Flfl^169–361,^ CENP-C, FBD, FBD^ΔFIM^ or GST-alone proteins were maintained on beads after immobilization and stored in PBS supplemented with 50% glycerol at −20 °C. His-tagged proteins were affinity purified on Ni-NTA resin (Qiagen) according to the manufacturer’s guide. After elution with 300 mM imidazole, His::TEV::Flfl^1–168^ and His::TEV::CENP-C^FBD^ were dialyzed against PBS for 4 h at 4 °C, and subsequently treated with His-tagged TEV protease (in house), re-incubated with Ni-NTA resin for 30 min on ice and supernatants were dialyzed against PBS+10% glycerol (PBSG) for 16 h at 4 °C and stored at −20 °C.

### Protein purification from cultured cells or embryos

We have previously published detailed protocols for sample preparation, protein A affinity purification and proteomic analysis in refs 45, 46. Briefly, ~10^9^ D.Mel-2 cells expressing protein A-tagged CENP-C, Flfl, Flfl^1–168^ or Flfl^169–973^ were lysed in 8 ml extraction buffer (EB; 50 mM HEPES pH 7.5, 100 mM CH_3_COOK, 100 mM NaCl, 50 mM KCl, 2 mM MgCl_2_, 2 mM EGTA-Na, 5 mM DTT, 0.5% NP-40, 5% glycerol and complete protease inhibitor cocktail) on ice using Power Gen 125 homogenizer (Fisher Scientific). Embryos (1 g) expressing protein A-tagged CENP-C or Flfl were homogenized in 8 ml EB on ice using Dounce tissue grinder (Wheaton). Homogenates were treated with 2,000 Kunitz units of DNase I (Sigma, D4263) for 5 min at 37 °C and 10 min at 25 °C and centrifuged (4 °C, 10 min, 10,000 *g*). Clarified lysates were mixed with rabbit immunoglobulin-G-conjugated Dynabeads (Life Technologies, 14302D) for 2 h at 4 °C, beads were washed four times in EB and proteins were eluted with 1 M NH_4_OH (10 min at 25 °C). Eluates were acetone precipitated and samples were analyzed by mass spectrometry.

Immunoaffinity purification of FLAG-tagged proteins was performed as follows: (1) Large scale: FLAG::CENP-C^FBD^, FLAG::CENP-C^WT^ or FLAG::CENP-C^ΔFIM^ were affinity purified from ~10^9^ stably transfected D.Mel-2 cells on anti-FLAGM_2_ magnetic beads (Sigma, M8823) as described above. Purified complexes were analyzed by immunoblotting or mass spectrometry. (2) Small-scale for the λ-PPase band-shift assay: stably transfected D.Mel-2 cells (1–5 × 10^6^ cells per well) expressing FLAG::CENP-C^FBD^ were treated with DMSO (carrier control), 50 nM okadaic acid (3 h at 25 °C) or double-stranded RNAs (dsRNAs) targeting *flfl*, *pp4c* or *kan* for RNA-mediated interference (RNAi). Cells were lysed in EB by passing a cell suspension through a G25 needle (five times) followed by centrifugation (4 °C, 10 min, 21,000 *g*). Supernatants were incubated with anti-FLAGM_2_ magnetic beads (4 °C, 3 h, 15 r.p.m.) and bound proteins were eluted with 1 M NH_4_OH (room temperature, 5 min), precipitated with ice-cold acetone and used in a λ-PPase assay.

Small-scale GFP purification was performed from 1–5 × 10^6^ D.Mel-2 cells expressing wild-type or ΔFIM variants of FLAG::CENP-C and transiently co-transfected with GFP-tagged Flfl, Flfl^1–168^ or Flfl^169–973^ (in pMT–GFP) on GFP–Trap beads (ChromoTek, gta) following the protocol detailed above (see small-scale FLAGM_2_ IP). Protein complexes were eluted with 1 × Laemmli sample buffer and analyzed by immunoblotting.

To pull down embryonic PP4, a wild-type (Oregon-R) syncytial embryo extract was incubated with GST (control) or GST::CENP-C^FBD^ (for 3 h at 4 °C, with gentle rotation) immobilized on Glutathione Sepharose 4b resin. The beads were then washed five times in EB, resuspended in Laemmli sample buffer, boiled and subjected to SDS–PAGE.

### Mass spectrometry

Mass spectrometric analyses of protein samples obtained after affinity purifications were performed at Mass Spectrometry Laboratory, Institute of Biochemistry and Biophysics, Polish Academy of Sciences (Warsaw, Poland)^21^. Samples were digested with trypsin (Promega V5111) and peptide mixtures were analyzed by liquid chromatography–tandem mass spectrometry (LC–MS/MS) using Nano-Acquity (Waters) LC system and Orbitrap Velos mass spectrometer (Thermo Electron Corp.). MS/MS raw data were analyzed by Mascot Distiller followed by Mascot Search (Matrix Science) against FlyBase database.

### *In vitro* complex formation

Recombinant Flfl^1–168^ or CENP-C^FBD^ were loaded (v=0.5 ml min^−1^; V=0.5 ml, respectively) onto a Superdex 200 size exclusion column (GE Healthcare), and individual fractions were analyzed by SDS–PAGE followed by Coomassie brilliant blue (CBB) staining. To reconstitute the dimeric complex *in vitro*, Flfl^1–168^ and CENP-C^FBD^ were mixed in an ~1:1 molar ratio, incubated on ice for 16 h, centrifuged (4 °C, 10 min, 21,000 *g*) and loaded onto the Superdex 200 column.

### IVTT and binding assays

For the interaction studies ^35^S-methionine-labelled Flfl, Flfl^1–168^, R2, PP4c, CENP-C-fl, CENPC-N′, CENPC-C′ or C1–C11 (see Fig. 1e) fragments of CENPC-C were expressed *in vitro* using the T_N_T T7 Quick Coupled Transcription/Translation System (Promega, L1170). Hundred nanograms of purified PCR fragments (C1–C11, Flfl^1–168^) or recombinant plasmids (Flfl, R2 or PP4c in pETDuet-1; CENP-C-fl, CENPC-N′ or CENPC-C′ in pDEST17) were added to a 30 μl reaction mixture (containing T_N_T Quick Master Mix, RNasin Plus RNase Inhibitor (Promega, N2611), T7 T_N_T PCR Enhancer, protease inhibitor cocktail and 0.5 MBq Methionine-L [^35^S] (Perkin Elmer, NEG709A001MC)), and incubated at 30 °C for 60 min. The mixture was centrifuged at 21,000 *g* at 25 °C for 5 min. The supernatant (IVTT input) was divided into equal parts and used for an *in vitro* binding assay in which GST only served as the negative control and other GST-tagged proteins were used as a bait. Bait proteins immobilized on Glutathione Sepharose 4b resin were resuspended in 800 μl of binding buffer (50 mM HEPES pH 7.4, 150 mM NaCl, 1 mM MgCl_2_, 1 mM EGTA, 1 mM DTT, 0.1% Triton X-100, complete protease inhibitor cocktail and 0.5 mg ml^−1^ BSA), mixed with IVTT-expressed ^35^S-Met-labelled prey and incubated for 1 h at 25 °C (with gentle rotation). Then beads were washed with washing buffer (binding buffer without BSA), transferred into new tubes and boiled in Laemmli sample buffer. Proteins were separated by SDS–PAGE and gels were then stained with Coomassie brilliant blue, dried and directly used for autoradiography. Exposure to hypersensitive film (Kodak BioMax MS film, 8222648) was carried out at −80 °C.

### Peptide array

Two peptide arrays were generated (Peptide Synthesis Laboratory, CR UK LRI, London): (1) interaction array: 82 different 20-mer peptides each shifted by 1 aa and covering the whole sequence of CENP-C^FBD^ were spotted onto a cellulose membrane; (2) substitution array: each and every residue in the 19-mer FIM motif (1048-PDESSADVVFKKPLAPAPR-1066) was substituted with 19 individual different aa giving 380 peptides in total, which were spotted onto a cellulose membrane. Membranes were activated by washing in 50% ethanol and 10% glacial acetic acid for 1 h and then washed three times in Buffer A (50 mM Tris, pH 7.5, 100 mM NaCl and 1 mM DTT) for 10 min each. Hundred nanomolar high purity untagged Flfl^1–168^ in Buffer A was left to incubate with the arrays at 4 °C overnight with continuous shaking. The membranes were then washed with Buffer A and incubated with anti-Flfl antibody in Buffer B (Buffer A supplemented with 0.1% Tween-20 and 5% milk powder) for 3 h at room temperature. After further washes, spots with bound Flfl^1–168^ were detected using goat anti-rat immunoglobulin-G–horseradish peroxidase–conjugated secondary antibody (2 h at room temperature) and visualized by chemiluminescence according to the manufacturer’s instruction (Merck Millipore, WBKLS0500).

### λ-PPase band-shift assay

Immunaffinity-purified FLAG::CENP-C^FBD^ and its phosphorylated forms were treated with exogenous λ-PPase (NEB, P0753): purified protein precipitates were resuspended in λ-PPase buffer supplemented with 1 mM MnCl_2_. Samples were divided into two equal portions and incubated at 30 °C in the presence or absence of λ-PPase for indicated times (max 2 h). The reaction was stopped by adding Laemmli sample buffer followed by heat inactivation (for 5 min at 95 °C). Samples were subjected to Phos-tag-containing SDS–PAGE.

### Phos-tag SDS–PAGE

For the better separation of phosphorylated FLAG::CENP-C^FBD^ species, protein samples were subjected to 15% SDS–PAGE containing 25 μM Phos-tag (Wako, AAL-107) in the presence of 70 μM MnCl_2._ Proteins were blotted to nitrocellulose membrane according to the manufacturer.

### Antibodies

Affinity-purified GST-tagged Flfl^N^, PP4c^1–50^ or CENP-C^1202–1411^ and R2::His were further purified by size exclusion chromatography (Superdex 75, GE Healthcare) in PBS and used as antigens to immunize rats (anti-Flfl and anti-CENP-C by IBMC, Portugal), a mouse (anti-PP4c by Harlan, UK) or a rabbit (anti-R2 by Harlan). The specificity of the antibodies was confirmed by immunoblotting (Supplementary Fig. 1a) after gene-specific RNAi in D.Mel-2 cells. The following antibodies were used in IB or IF experiments: rat anti-Flfl serum (IB: 1:10,000, IF: 1,000), mouse anti-PP4c serum (IB: 1:3,000), rat anti-CENP-C serum (IB: 1:3,000, IF: 1:1,000), mouse anti-FLAGM_2_ (Sigma, F3165; IB: 1:1,0000, IF: 1:5,000), mouse anti-αTubulin (clone DM1A; Sigma, T9026; IB: 1:10,000, IF: 1:500) and mouse anti-GFP (Roche, 11814460001; IB: 1:2,000, IF:1:1,000). Chicken anti-CID purified antibody (IF: 1:2,000), rabbit anti-Mis12 serum (IF: 1:500), rabbit anti-Nsl1 serum (IF: 1:1,000), sheep anti-Spc105 serum (IF: 1:1,000), rabbit anti-Ndc80 serum (1:500) and rabbit anti-Spd2 (IF: 1:2,000) antibodies were generated and used in previous studies in our laboratory^21^,^47^,^48^. Secondary antibodies for IB or IF were obtained from Jackson ImmunoResearch (horseradish peroxidase or DyLight conjugates) or Life Technologies (AlexaFluor conjugates) and used at 1:10,000 (IB) or 1:500 (IF) dilution.

### Transgenic flies

Transgenic *Drosophila melanogaster* stocks constitutively expressing protein A-tagged CENP-C (*w; P(maternal αtub-Cenp-C::PrA)*) or Flfl (*w; +; P(maternal αtub-PrA::Flfl)*) in female germline were made using standard P-element transformation (Fly Facility, Department of Genetics, University of Cambridge). Fly stocks were cultured at 25 °C on standard Drosophila food. Fly stocks expressing the transgenic proteins in a comparable (same or lower) level than that of the endogenous protein were used in proteomic studies.

### Cell cultures

D.Mel-2 cells (Life Technologies) were grown in Express Five SFM medium (Life Technologies, 10486-025) supplemented with 2 mM L-glutamine (25030-024) and Pen Strep (15140-122) according to standard procedures. Protein A::Flfl, protein A::Flfl^1–168^, protein A::Flfl^169–973^, CENP-C::protein A, FLAG::CENP-C^FBD^, FLAG::CENP-C^WT^, FLAG::CENP-C^ΔFIM^, Flfl::FLAG, FLAG::Flfl^1–168^, GFP::CENP-C^WT^/FLAG::Flfl^1–168^, GFP::CENP-C^ΔFIM^/FLAG::Flfl^1–168^, FLAG::PP4c^WT^ and FLAG::PP4c^PhD^ stable cell lines were established using FuGENE HD Transfection Reagent (Promega, E2311) following standard procedures^21^. Briefly, 3 μg of DNA was mixed with 15 μl of the reagent in 150 μl of nuclease-free water for each transfection mixture and incubated at room temperature for 15 min. Then, each mixture was added drop wise to a well of a six-well plate, where cells were previously seeded in 2 ml of medium at 40–60% confluency. The antibiotic selection started 48 h post-transfection and was carried out according to standard procedures^45^.

### RNAi and immunostaining of cells

The sequences of primers used to amplify dsRNAs for RNAi experiments are given in Supplementary Table 7. For Flfl, PP4c and CENP-C more than one dsRNA was generated. We have not noticed differences between them, in terms of effectiveness of the knockdown or a phenotype. The time for maximal protein depletion was empirically determined; in general the most severe phenotype was attained after two sequential rounds of RNAi. Cells were transfected with dsRNAs using TransFast (Promega, E2431) and then plated on non-treated or 0.5 mg ml^−1^ concanavalin-A-coated cover slips, fixed with 4% formaldehyde and immunostained^47^,^48^. Briefly, fixed cells were blocked in 3% BSA, 0.5% Triton X-100 in PBS, then incubated in primary antibody-containing PBT (1% BSA, 0.1% Triton X-100 in PBS) for 2–4 h, followed by three washes in PBT, 1 h-long incubation in secondary antibody-containing PBT and three more washes in PBT. After this, cover slips were rinsed with pure water and mounted on slides with ProLong Gold antifade reagent (Life Technologies, P36934).

### Microscopy

Images were acquired on a Zeiss Axiovert 200 M microscope (objective × 100/numerical aperture 1.4) with a Cool-SNAP HQ camera (Photometrics) using MAG Biosystems Software—Metamorph (Molecular Devices). Images showing Flfl, Flfl::FLAG or FLAG::Flfl^1–168^ localization were acquired using a Zeiss LSM 510 Meta Confocal Microscope (× 100 objective) using LSM510 software (release version 4.2 SP1, Carl Zeiss MicroImaging) and processed using ImageJ (NIH, USA). Line scans were done using ImageJ and values were imported to MS Excel, which was used to prepare plots.

### Phenotypic analysis

The CENP-C displacement phenotype was most reliably scored in cells in which the spindle axis was parallel to the cover slip with well separated centrosomes, as shown by Spd2 staining, and where the chromosomes were largely congressed at the equator (Fig. 4a). This prevented false positives due to centrosome proximal to centromeric signals. It is therefore likely that our phenotypic counts are underestimates of the penetrance of the defect. Data from four independent RNAi experiments were analyzed using GraphPad Prism software to produce the graph in Fig. 4b. The expressions of wild-type and ΔFIM versions of CENP-C were assessed after four RNAi experiments (Fig. 4c) and wild-type and PhD versions of PP4c after two RNAi experiments (Fig. 4d). A minimum of 50 mitotic cells were scored for each variant within each experiment. In experiments, which involved replacement of endogenous CENP-C with the transgenic CENP-C constructs, only those cells were scored that showed normal levels of CENP-C.

### Crystallographic methods

Crystals of native protein–peptide complex were grown in sitting drops by the vapour diffusion method^49^. Solution of protein at 22 mg ml^−1^ was incubated overnight with peptide (PDESSADVVFKKPLAPAPR) in a ratio of 1:1.3. Crystals of complex were grown by mixing 0.2 μl of this solution with a 1:1 dilution of ½ reservoir buffer (3.31 M Ammonium Sulfate, 9.5% Glycerol) and ½ Silver Bullet screen (Hampton Research; 0.2% w/v 4-Aminobenzoic, 0.2% w/v Azelaic acid, 0.2% w/v o-Sulfobenzoic acid monoammonium salt, 0.2% w/v p-Coumaric acid, 0.2% w/v Sodium 4-aminosalicylate dihydrate and 20 mM HEPES sodium pH 6.8) with distilled water. Crystals grew in space group P6_1_ (unit cell dimensions: *a*=*b=*67.75 Å, *c=*53.37 Å) to a size of 0.4 × 0.2 × 0.1 mm. Under the assumption of one protein molecule per asymmetric unit, a *V*_M_ value of 2.46 Å^3^* *Da^−1^ corresponding to a solvent content of 49.9% was calculated^50^. Crystals used for data collection were soaked in 3.4 M sodium malonate for a period of 2 min, transferred to loops and immersed in liquid nitrogen. X-ray diffraction data were collected at beamline ID29 (see ref. 51) of the European Synchrotron Radiation Facility. Data used to solve the structure were collected from a single crystal flash frozen at −180 °C. All data were processed and scaled using XDS^52^. Data collection statistics are shown in Table 3.

The structure of Flfl^1–122^ was solved by molecular replacement using the programme Mr Bump^53^ and two structures of RanBD and Spred-1 domains (PDB i.d. 1XOD, 1RRP) with a sequence identity of 20% as search models in a Phaser ensemble. The structure was iteratively rebuilt using Coot^54^ and refined with Refmac5 (see ref. 55) and phenix.refine^56^. The peptide sequence was docked after refinement for Flfl^1–122^ alone had converged, and anisotropic B-factor parameterization was employed for all non-water atoms. The structure was validated using tools from the Molprobity suite^57^, which showed 99% of residues to be in the favoured Ramachandran region and gave an overall clash score of 0.52.

### Image processing

Scanned autoradiographs, gels and immunoblots were cropped and compiled into figures using Adobe Photoshop and Illustrator CS6. Uncropped scans are provided in Supplementary Figs 5–9.

## Author contributions

Z.L. performed interaction and *in vitro* assays, protein purifications from cultured cells and embryos, proteomic analysis, mutagenesis and antibody production. S.L. and M.R.S. undertook crystallographic experiments. M.S.S. contributed to the phenotypic analysis of cultured cells. M.R.P. performed protein purifications from cultured cells, proteomic analysis and cell biology experiments. Z.L., M.R.P., M.R.S. and D.M.G. planned the experiments and wrote the paper that was discussed by all authors.

## Additional information

**How to cite this article:** Lipinszki, Z. *et al*. Centromeric binding and activity of Protein Phosphatase 4. *Nat. Commun.* 6:5894 doi: 10.1038/ncomms6894 (2015).

**Accession code:** The atomic coordinates and structure factors have been deposited in the Protein Data Bank under accession code 4WSF.

## Supplementary Material

Supplementary Information Supplementary Figures 1-9, Supplementary Tables 1-7 and Supplementary Reference.

## Figures and Tables

**Table 1:** Falafel-interacting proteins in D.Mel-2 cells.

**Protein A::Falafel affinity purification**				
**#**	**FlyBase CG**	**Protein**	**Score**	**Coverage (%)**
1	9351	Falafel (bait)	11,959	26
2	31258	CENP-C	1,178	13
3	42389	CG42389	1,124	10
4	32505	PP4c	872	27
5	2890	R2	316	7

AP-MS, affinity purification-coupled mass spectrometry.

AP-MS reveals protein A::Falafel pulling down centromeric protein CENP-C as well as R2 and PP4c, subunits of PP4, from D.Mel-2 cells with good Mascot scores and sequence representation (coverage).

**Table 2:** CENP-C-interacting proteins in D.Mel-2 cells.

**CENP-C::protein A affinity purification**				
**#**	**FlyBase CG**	**Protein**	**Score**	**Coverage (%)**
1	31258	CENP-C (bait)	6,141	31
2	9351	Falafel	705	16
3	32505	PP4c	632	28
4	2890	R2	621	23
5	17870	14-3-3zeta	268	18
6	31196	14-3-3epsilon	239	24
7	13329	CENP-A/CID	184	12
8	4817	SSRP1 (FACT complex)	98	3
9	10223	Topoisomerase2	86	1
10	1828	Dre4 (FACT complex)	53	1

AP-MS, affinity purification-coupled mass spectrometry.

AP-MS of CENP-C::protein A reveals all three subunits of PP4 interacting with CENP-C in D.Mel-2 cells.

**Table 3:** Data collection and refinement statistics (molecular replacement).

	**Falafel (1–122)_CENP-C (FIM)**
Data collection	
Space group	P6_1_
Cell dimensions	
*a*, *b*, *c* (Å)	67.75, 67.75, 53.37
*α*, *β*, *γ* (°)	90, 90, 120
Resolution (Å)	58.68–1.50 (1.55–1.50)
*R*_*sym*_ or *R*_*merge*_	0.03 (0.39)
*I/σI*	11.72 (1.84)
Completeness (%)	99.70 (98.19)
Redundancy	6.7 (6.45)
	
Refinement	
Resolution (Å)	58.68–1.50
No. reflections	22,313
*R*_work_*/R*_free_	0.13 (0.19)/0.17 (0.26)
No. atoms	1,061
Protein	971
Ligand/ion	5
Water	85
B-factors	31.5
Protein	30.6
Ligand/ion	44.6
Water	41.6
R.m.s deviations:	
Bond lengths (Å)	0.009
Bond angles (°)	1.17

FIM, Falafel-interacting motif; r.m.s, root mean square.

Statistics for the highest-resolution shell are shown in parentheses.
