# Supplementary material for: Centromeric binding and activity of Protein Phosphatase 4
Source: Nat Commun. 2015 Jan 6;6:5894. doi: 10.1038/ncomms6894 (PMC4354016; doi:10.1038/ncomms6894)
Supplement: Supplementary Information — Supplementary Figures 1-9, Supplementary Tables 1-7 and Supplementary Reference. [file ncomms6894-s1.pdf]

Supplementary Figure 1

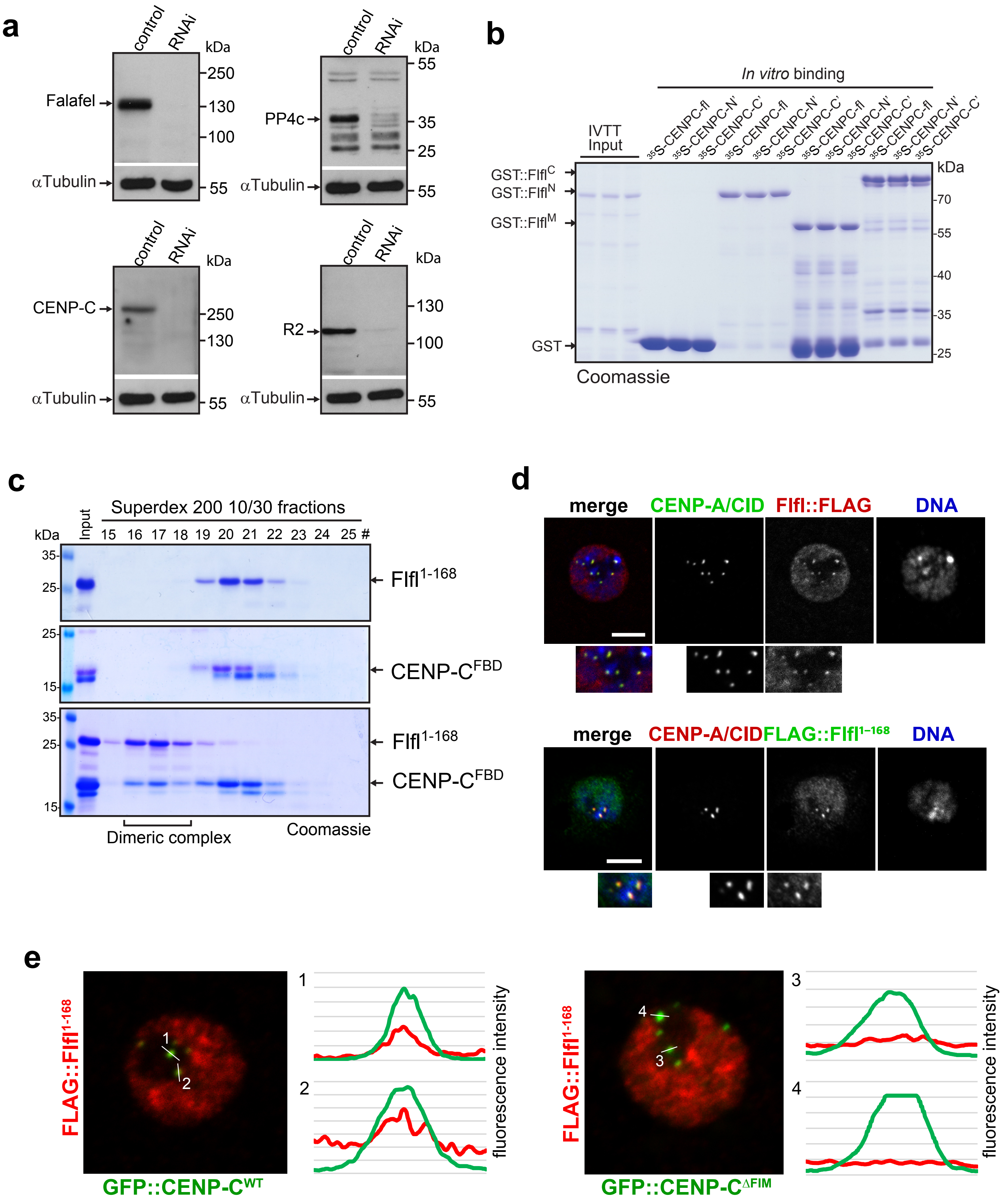

Supplementary Figure 1. Protein phosphatase 4 (PP4) interacts directly with CENP-C via its regulatory 3-type subunit, Falafel.

(a) Western-blots confirming the specificity of anti-Falafel, anti-PP4c, anti-R2 and anti-CENP-C antibodies made and used in this study. Here the antibodies are used to probe extracts of control dsRNA-treated cells (“control”) and cells transfected with dsRNAs directed against the respective proteins (“RNAi”). Anti-αTubulin provides a loading control. **(b)** Samples corresponding to the autoradiograph shown in **Fig. 1d** were run on a more concentrated SDS-PAGE (to reveal the ~25 kDa GST) and stained with Coomassie Brilliant Blue to demonstrate the loading of recombinant fragments of Falafel as well as GST used in the binding assay with ^{35}S-Met-labelled CENPC-fl, CENPC-N’ or CENPC-C’. **(c)** SDS-PAGE analysis of size exclusion chromatography fractions of the Flfl$^{1\text{-}168}$ and CENP-C^{FBD} fragments run either alone (upper two panels) or as combined mixture (lower panel). This shows that Flfl$^{1\text{-}168}$ and CENP-C^{FBD} form a stable complex *in vitro*. Input: affinity purified recombinant proteins before chromatography. **(d)** Confocal microscope images of immunostained cultured cells expressing Falafel::FLAG (Flfl::FLAG) or FLAG::Flfl$^{1\text{-}168}$. Both Falafel::FLAG and FLAG::Flfl$^{1\text{-}168}$ co-localize with CENP-A/CID. Magnified insets below main panels show FLAG and CENP-A/CID signals co-localized at centromeres. This result further supports our conclusion that Flfl$^{1\text{-}168}$ is necessary and sufficient for CENP-C binding, both *in vitro* and *in vivo*. **(e)** Line scans across kinetochore area in cells co-expressing either FLAG::Flfl$^{1\text{-}168}$ and GFP::CENP-C^{WT} (left panel) or FLAG::Flfl$^{1\text{-}168}$ and GFP::CENP-C$^{\Delta FIM}$ (right panel). Fluorescence intensity measurements along the lines drawn across kinetochores (numbers 1 through 4) show that FLAG::Flfl$^{1\text{-}168}$ co-localizes with GFP::CENP-C^{WT}, but not with GFP::CENP-C$^{\Delta FIM}$. Flfl$^{1\text{-}168}$ fusion is in red and CENP-C transgenes are in green, and line colors on line scans match the fluorophores used for cell staining; examples shown here are exactly the same cells as in **Figure 2d**.

Supplementary Figure 2

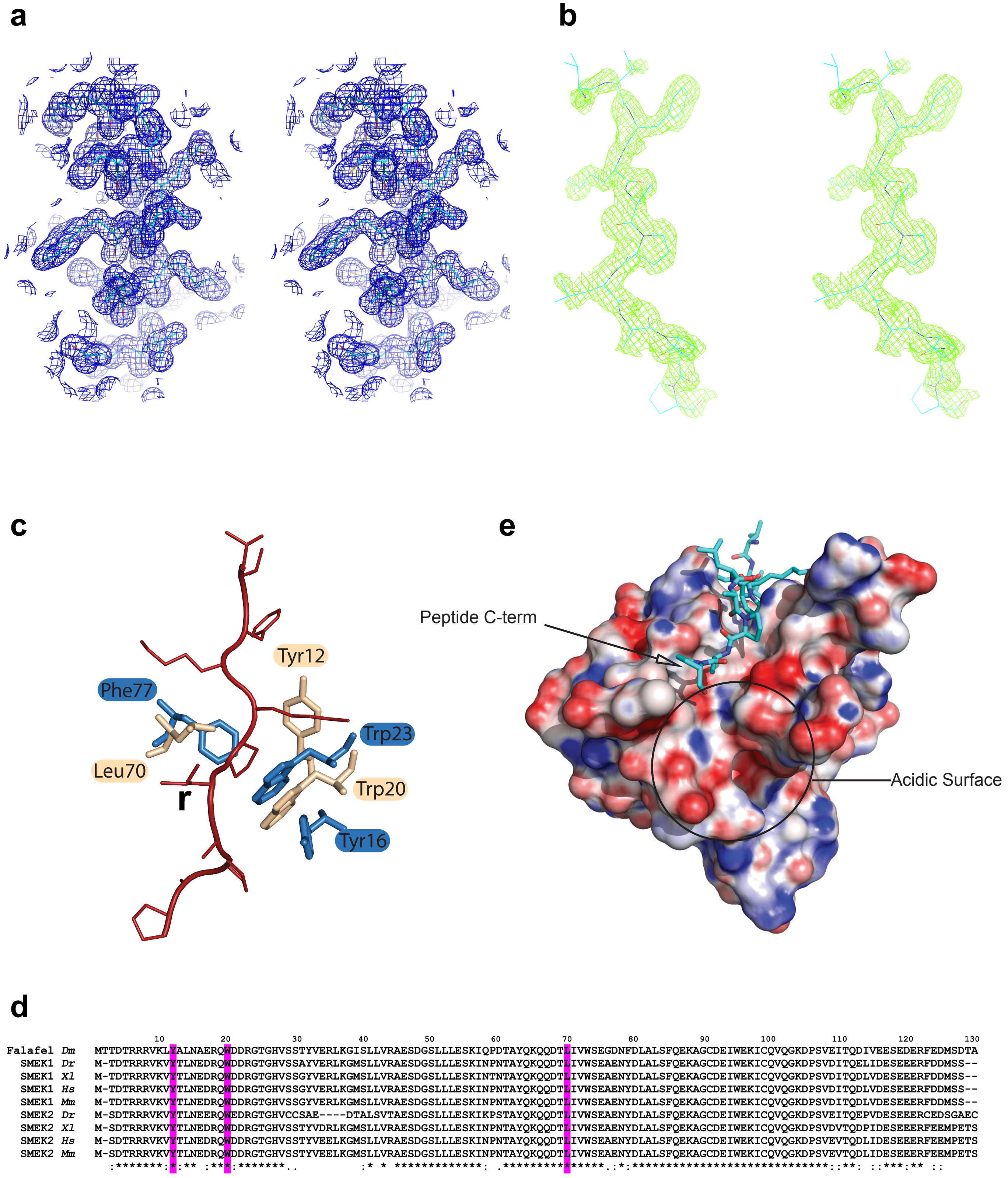

Supplementary Figure 2. Structural analysis of Falafel - CENP-C interactions

(a) Stereo view of the final refined 2Fo-Fc map (blue) of the EVH1 domain contoured at 1σ. **(b)** Stereo view of unbiased omit map (green) of the CENP-C peptide contoured at 2σ plotted over final refined atomic coordinates. **(c)** Superposition of Mena EVH1 (blue) and Flfl$^{1\text{-}122}$ (wheat) side chains involved in protein peptide binding. The differing orientations of the strongly conserved Tyr12 (Flfl$^{1\text{-}122}$) and Tyr16 (EVH1) can be clearly seen. **(d)** Alignment of R3-type subunits from *Drosophila* (*Dm*), human (*Hs*), mouse (*Mm*), fish (*Dr*) and frog (*Xl*) showing the level of sequence conservation within EVH1 domains. Asterisks indicate identical residues. Three amino acids critical for CENP-C binding to Falafel, Tyr 12, Trp20 and Leu70 (labelled in purple), are absolutely conserved among listed species. **(e)** Electrostatic surface of Flfl$^{1\text{-}122}$ coloured from blue (positively charged) to red (negatively charged) with the FIM peptide represented as sticks. The groove formed by Flfl$^{1\text{-}122}$ surface found at the C-terminus of FIM is negatively charged (encircled "Acidic surface") and could accommodate with the basic residues following Pro1065 in CENP-C protein. This suggests that the reverse orientation of CENP-C^{FIM} would be both sterically and electrostatically disfavoured and therefore we are confident that the crystal structure shows the correct peptide direction.

Supplementary Figure 3

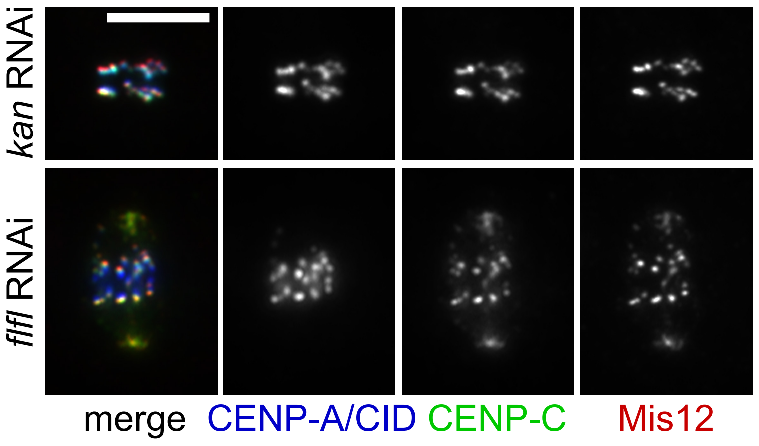

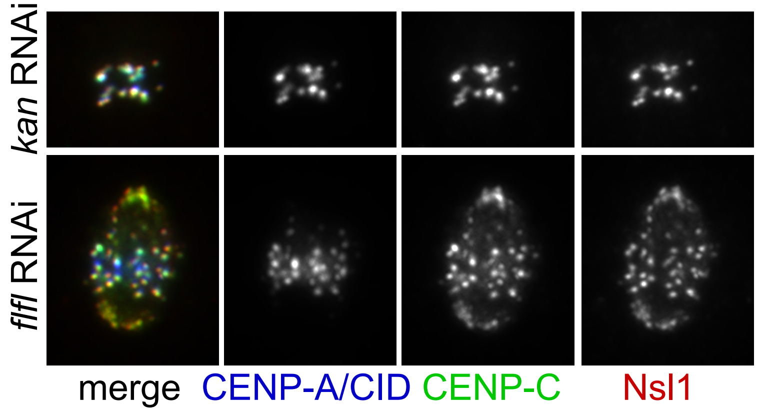

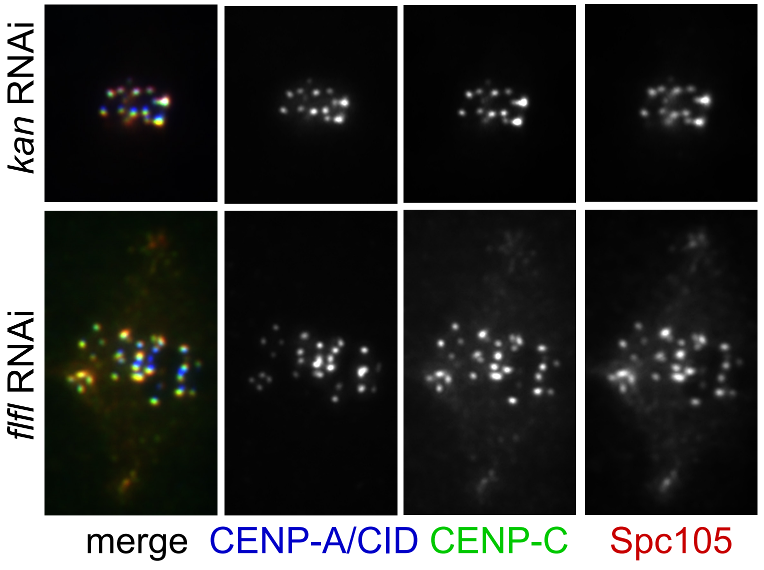

Supplementary Figure 3. The association between PP4 and CENP-C is required for kinetochore integrity

Fluorescence microscopy of cultured D.Mel-2 cells treated with control (*kan*) or *flfl*-targeting dsRNAs and subsequently immunostained to reveal CENP-A/CID, CENP-C and different KMN network proteins: Mis12 (upper panels), Nsl1 (middle panels) or Spc105 (lower panels). Core kinetochore proteins perfectly follow CENP-C mislocalization onto the spindle and spindle poles in *flfl*-depleted mitotic cells whereas the localization of CENP-A/CID is unaffected. Scale bar is 5 μm.

Supplementary Figure 4

a

```
PP4c   ---MSDYSDLDRQIEQLKRCEIIKENEVKALCAKAREILVEEGNVQRVDSPVTVCGDIHG   57
PP2A   MEDKATTKDLDQWIEQLNECNQLTETQVRTLCDKAKEILSKESNVQEVKCPVTVCGDVHG   60
          :  .***: ****:.*: :.*.:*::** **:*** :*.***.*..*******:**

PP4c   QFYDLKELFKVGGDVPEKNYLFMGDFVDRGYYSVETFLLLLALKVRYPDRITLIRGNHES  117
PP2A   QFHDLMELFRIGGKSPDTNYLFMGDYVDRGYYSVETVTLLVALKVRYRERITILRGNHES  120
       **:** ***::**. *:.*******:**********. **:****** :***::******
                                  D85N                        H115N
```

b

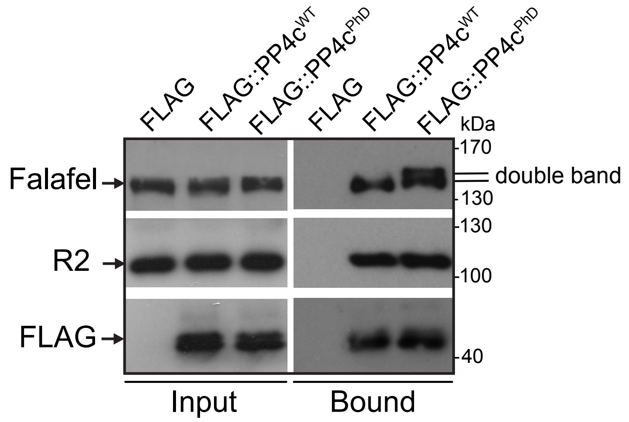

c

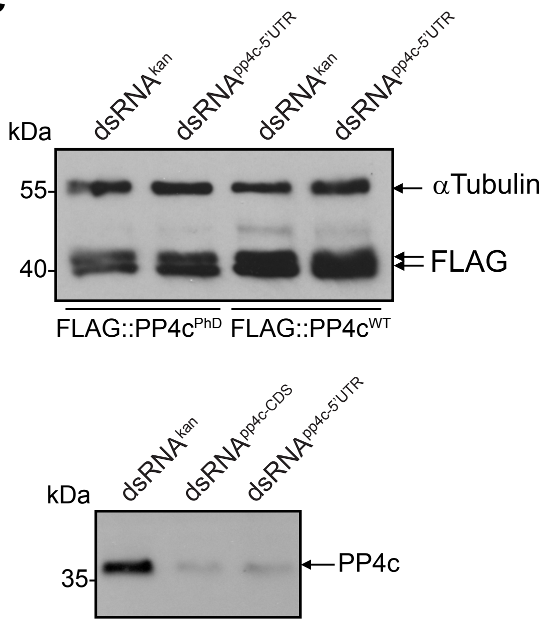

Supplementary Figure 4. PP4c^{PhD} is catalytically inactive, but structurally intact and forms a trimeric complex with Falafel and R2

(a) Protein sequence alignment of the catalytic subunits of *Drosophila* PP4 (PP4c, first 117 residues) and PP2A (first 120 residues) revealing the conserved amino acids of the active centers based on the work by Myles and colleagues[1]. Residues D85 and H115 of PP4c were mutated to N85 and N115 in the full length protein, which then was used as a Phosphatase dead mutant (PP4c^{PhD}) in this study. **(b)** Anti-FLAG immunoprecipitation of FLAG alone, FLAG::PP4c^{WT} or FLAG::PP4c^{PhD} from stable cell lines. Western-blots are probed with antibodies to reveal Falafel, R2 or FLAG. This shows that FLAG::PP4c^{PhD} is structurally intact and forms a trimeric complex with Falafel and R2. Interestingly, it also binds to the phosphorylated form of Falafel ("double band"). **(c)** Western-blot (top panel) showing comparable expression levels of FLAG::PP4c^{WT} and FLAG::PP4c^{PhD} in two respective cell lines treated with control (dsRNA^{kan}) or *pp4c* 5'UTR-targeted (dsRNA$^{pp4c\text{-}5'UTR}$) interfering dsRNAs. αTubulin serves as loading control. The bottom panel compares the efficiency of RNAi when D.Mel-2 cells were transfected with dsRNA^{kan} (negative control), dsRNA$^{pp4c\text{-}CDS}$ (dsRNA targeting the PP4c coding sequence) or *dsRNA*$^{pp4c\text{-}5'UTR}$.

Supplementary Figure 5

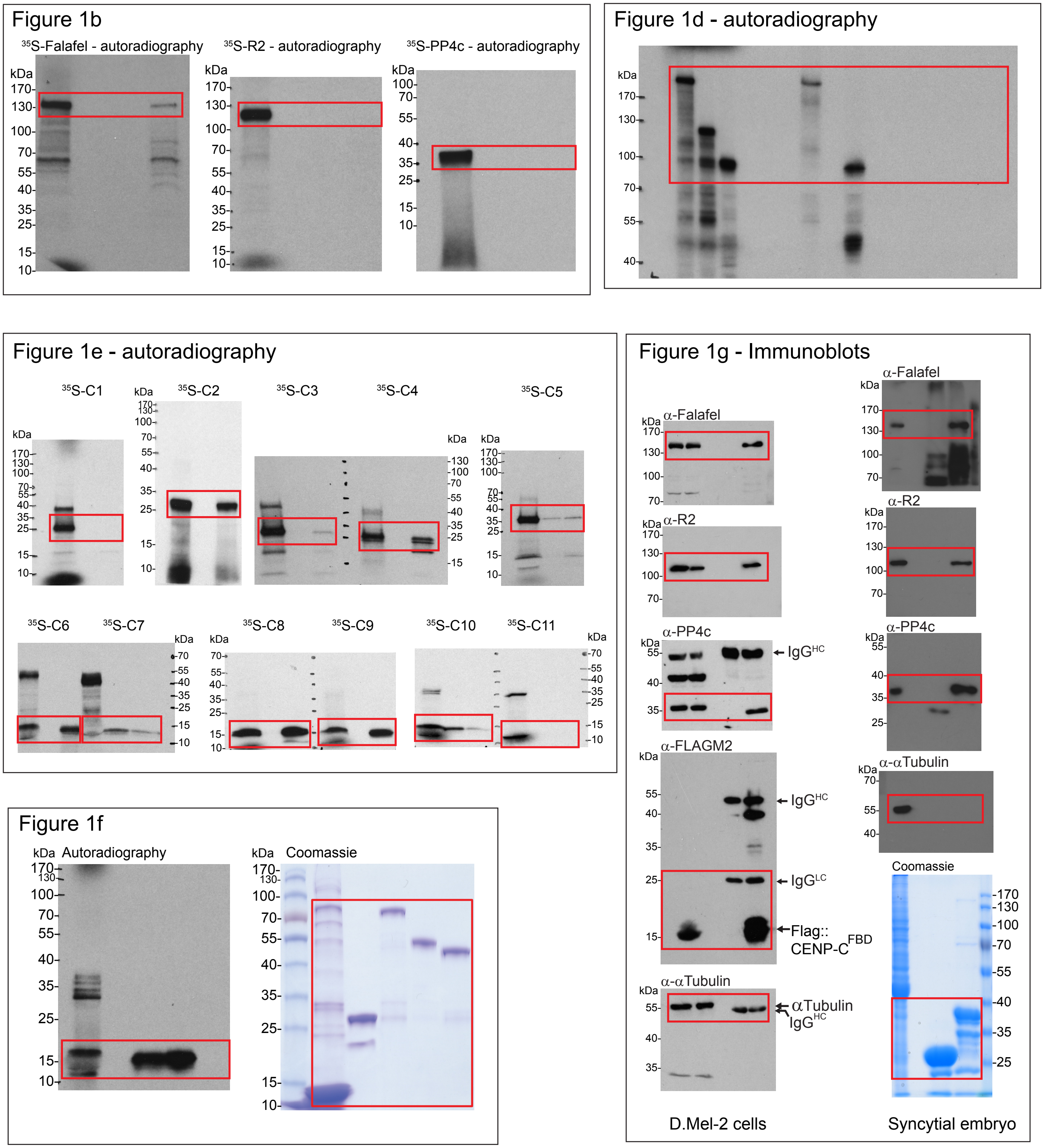

Supplementary Figure 5. Uncropped scanned images of immunoblots, autoradiograms and stained gels used in Figure 1.

The cropped regions are indicated by red boxes.

Supplementary Figure 6

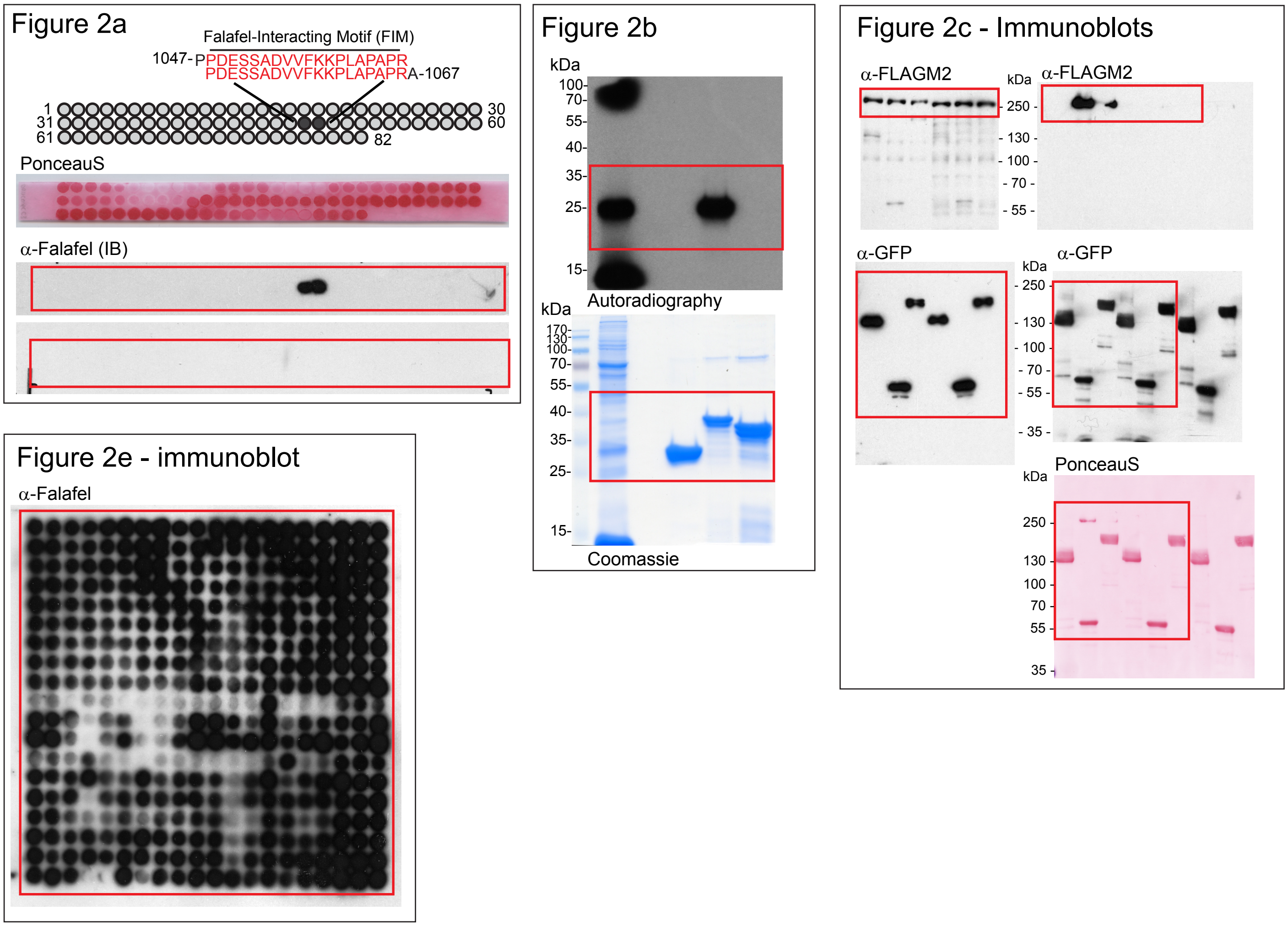

Supplementary Figure 6. Uncropped scanned images of immunoblots, an autoradiogram and a stained gel or nitrocellulose membranes used in Figure 2.

The cropped regions are indicated by red boxes.

Supplementary Figure 7

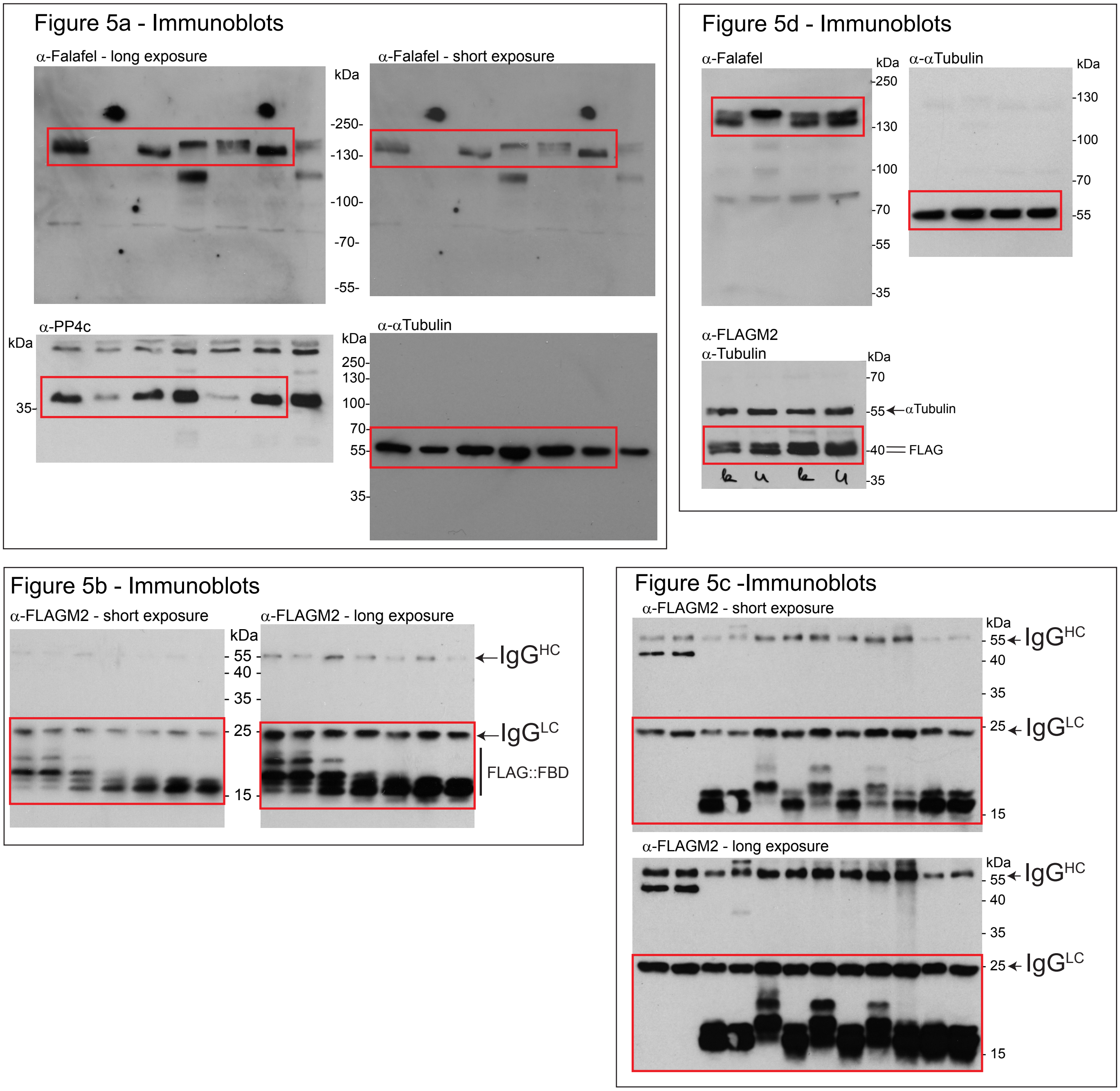

Supplementary Figure 7. Uncropped scanned images of immunoblots used in Figure 5.

The cropped regions are indicated by red boxes.

Supplementary Figure 8

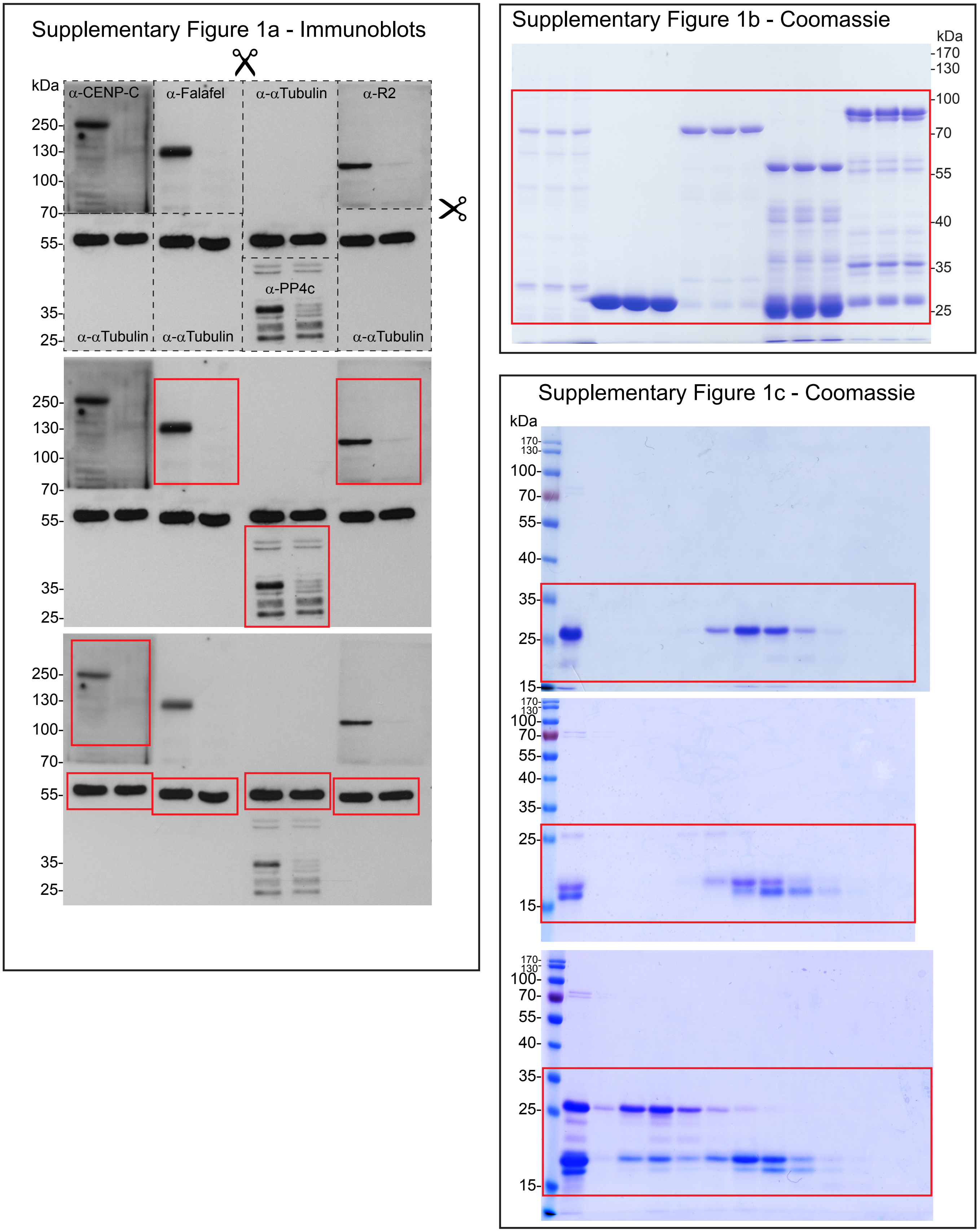

Supplementary Figure 8. Uncropped scanned images of immunoblots and stained gels used in Supplementary Figure 1.

The cropped regions are indicated by red boxes. Dashed line boxes (black) indicate where the nitrocellulose membrane was cut before being subjected to immunoblotting to reveal the antigens indicated.

Supplementary Figure 9

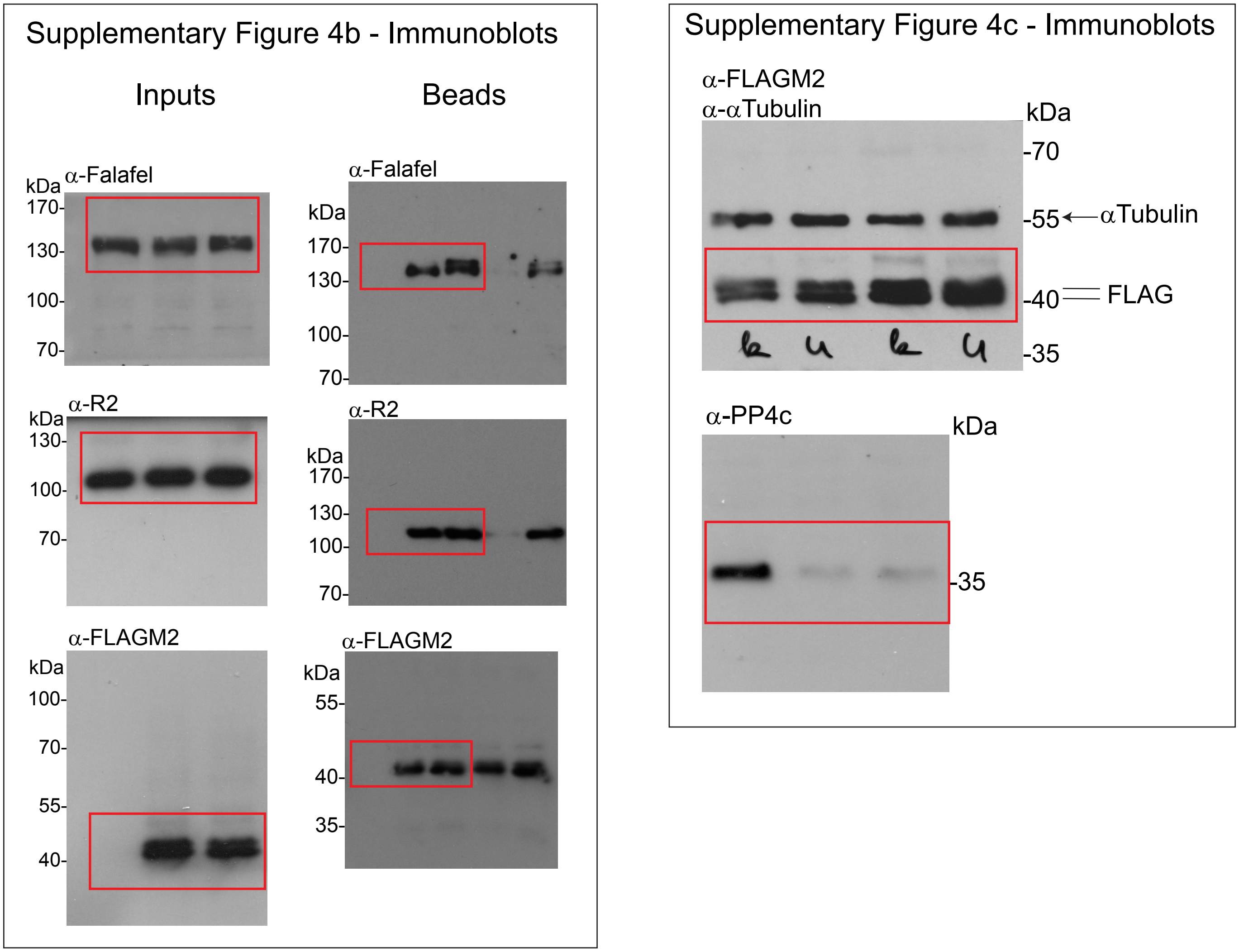

Supplementary Figure 9. Uncropped scanned images of immunoblots used in Supplementary Figure 4.

The cropped regions are indicated by red boxes.

SUPPLEMENTARY TABLES

Supplementary Table 1. Protein A::Falafel interacting proteins purified from *Drosophila* embryos

**Protein A::Falafel affinity purification from *Drosophila* embryo extract**				
**#**	**FlyBase CG**	**Protein**	**Score**	**Coverage %**
1	2890	**R2**	4830	55
2	9351	**Falafel [bait]**	3965	30
3	32505	**PP4c**	3096	57
4	6664	CG6664	379	24
5	31258	CENP-C	155	2

Affinity purification-coupled mass spectrometry (AP-MS) showing proteins associated with Protein A::Falafel expressed in *Drosophila* syncytial embryos. These include CENP-C and other PP4 subunits: R2 and PP4c. Mascot scores and protein sequence coverage are shown in separate columns.

Supplementary Table 2. CENP-C::protein A interacting proteins purified from *Drosophila* embryos

CENP-C::protein A affinity purification from *Drosophila* embryo extract				
**#**	**FlyBase CG**	**Protein**	**Score**	**Coverage %**
1	31258	**CENP-C [bait]**	6502	56
2	11451	Spc105/KNL1	250	2
3	17383	JIGR-1	182	22
4	2890	**R2**	142	6
5	9351	**Falafel**	131	6
6	5393	Apontic/TDF	100	12
7	6386	NHK-1/VRK	68	2
8	1558	Nsl1	65	7
9	18608	PROD	62	2
10	32505	**PP4c**	61	3

AP-MS of proteins associated with CENP-C::protein A expressed in *Drosophila* syncytial embryos. These include the Falafel, R2 and PP4c, subunits of the PP4 holoenzyme as well as known interactors of CENP-C.

Supplementary Table 3. Protein A::Flfl$^{1\text{-}168}$ co-purifies CENP-C from *Drosophila* cultured cells

Protein A::Flfl$^{1\text{-}168}$ affinity purification from cell extract				
**#**	**FlyBase CG**	**Protein**	**Score**	**Coverage %**
1	9351	**Falafel [bait]**	824	43
2	31258	CENP-C	228	3

AP-MS of Protein A::Flfl$^{1\text{-}168}$ expressed in cultured D.Mel-2 cells showing the co-purification of endogenous CENP-C. Mascot scores and protein sequence coverage are shown in separate columns.

Supplementary Table 4. Protein A::Flfl$^{169\text{-}973}$ does not interact with CENP-C in *Drosophila* cultured cells

Protein A:: Flfl$^{169\text{-}973}$ affinity purification from cell extract				
**#**	**FlyBase CG**	**Protein**	**Score**	**Coverage %**
1	9351	**Falafel [bait]**	4545	29
2	31258	CENP-C	0	0

AP-MS of Protein A::Flfl$^{169\text{-}973}$ expressed in cultured DMel-2 cells showing no interaction with endogenous CENP-C. Mascot scores and protein sequence coverage are shown in separate columns.

Supplementary Table 5. FLAG::CENP-C^{WT} co-purifies PP4 from *Drosophila* cultured cells

FLAG::CENP-C^{WT} affinity purification from cell extract				
**#**	**FlyBase CG**	**Protein**	**Score**	**Coverage %**
1	31258	**CENP-C [bait]**	54276	61
2	9351	**Falafel**	647	16
3	2890	**R2**	680	26
4	32505	**PP4c**	347	34

AP-MS of FLAG::CENP-C^{WT} expressed in cultured D.Mel-2 cells showing the co-purification of the entire PP4 holoenzyme. Mascot scores and protein sequence coverage are shown in separate columns.

Supplementary Table 6. FLAG::CENP-C$^{\Delta FIM}$ does not interact with PP4 in *Drosophila* cultured cells

FLAG::CENP-C$^{\Delta FIM}$ affinity purification from cell extract				
**#**	**FlyBase CG**	**Protein**	**Score**	**Coverage %**
1	31258	**CENP-C [bait]**	44375	58
2	9351	**Falafel**	0	0
3	2890	**R2**	0	0
4	32505	**PP4c**	0	0

AP-MS of FLAG::CENP-C$^{\Delta FIM}$ expressed in cultured D.Mel-2 cells showing none of the PP4 subunits co-purifying with the bait protein. Mascot scores and protein sequence coverage are shown in separate columns.

Supplementary Table 7. Oligonucleotide primers used in this study (5’-3’)

Gateway cloning:	Primer sequence (5'-3')
GW-Falafel-Fw	GGGGACAAGTTTGTACAAAAAAGCAGGCTTAATGACGACTGACACCCGCCGACGCG
GW-Falafel-Rev	GGGGACCACTTTGTACAAGAAAGCTGGGTACTATGCCTGACGCGCGCGCTTTTGT
GW-PP4c-Fw	GGGGACAAGTTTGTACAAAAAAGCAGGCTTAATGTCCGACTACAGCGACCTGGACC
GW-PP4c-Rev	GGGGACCACTTTGTACAAGAAAGCTGGGTATTAGAGAAAGTAGTCCGCCTGAGGC
GW-R2-Fw	GGGGACAAGTTTGTACAAAAAAGCAGGCTTAATGGTCACCATGGAAAACAGCGACG
GW-R2-Rev	GGGGACCACTTTGTACAAGAAAGCTGGGTACTACTGCATCATCACCTCCTGGCTG
GW-Flfl-1-361-Fw	GGGGACAAGTTTGTACAAAAAAGCAGGCTTAATGACGACTGACACCCGCCGACGCG
GW-Flfl-1-361-Rev	GGGGACCACTTTGTACAAGAAAGCTGGGTAGAGAGTTTTGTAGAACGAGTCCTTG
GW-Flfl-362-666-Fw	GGGGACAAGTTTGTACAAAAAAGCAGGCTTAACCTGCCTAGGCATTCTGCAGGCGC
GW-Flfl-362-666-Rev	GGGGACCACTTTGTACAAGAAAGCTGGGTACATTTCCTCTTCCTCCTCCATTTGC
GW-Flfl-667-973-Fw	GGGGACAAGTTTGTACAAAAAAGCAGGCTTATGGTTTAACGAGGAGGACGATTTCA
GW-Flfl-667-973-Rev	GGGGACCACTTTGTACAAGAAAGCTGGGTACTATGCCTGACGCGCGCGCTTTTGT
GW-Flfl-1-168-Fw	GGGGACAAGTTTGTACAAAAAAGCAGGCTTAATGACGACTGACACCCGCCGACGCG
GW-Flfl-1-168-Rev	GGGGACCACTTTGTACAAGAAAGCTGGGTACTACTCCAGAGCCATCGACAACTTTTCC
GW-Flfl-169-361-Fw	GGGGACAAGTTTGTACAAAAAAGCAGGCTTAATGTCGGAGAGCTATATCAAGAAGC

GW-Flfl-169-361-Rev	GGGGACCACTTTGTACAAGAAAGCTGGGTACTAGAGAGTTTTGTAGAACGA GTCCTTG
GW-Flfl-169-973-Fw	GGGGACAAGTTTGTACAAAAAAGCAGGCTTAATGTCGGAGAGCTATATCAA GAAGC
GW-Flfl-169-973-Rev	GGGGACCACTTTGTACAAGAAAGCTGGGTACTATGCCTGACGCGCGCTTT TGT
GW-PP4c-1-50-Fw	GGGGACAAGTTTGTACAAAAAAGCAGGCTTAATGTCCGACTACAGCGACCT GGACC
GW-PP4c-1-50-Rev	GGGGACCACTTTGTACAAGAAAGCTGGGTATTAGGTCACTGGCGAGTCCAC ACGCTGC
GW-CENP-C-Fw	GGGGACAAGTTTGTACAAAAAAGCAGGCTTAATGTCGAAGCCCCAGAACAA CGACA
GW-CENP-C-Rev	GGGGACCACTTTGTACAAGAAAGCTGGGTACTAACTGCGTATACACATCAG CACACTG
GW-CENP-C-N-Fw	GGGGACAAGTTTGTACAAAAAAGCAGGCTTAATGTCGAAGCCCCAGAACAA CGACA
GW-CENP-C-N-Rev	GGGGACCACTTTGTACAAGAAAGCTGGGTAATTACAGTTCGTTCTCCATCGC CCT
GW-CENP-C-C-Fw	GGGGACAAGTTTGTACAAAAAAGCAGGCTTAATGGTACCACCTCCTATCGA ATATGT
GW-CENP-C-C-Rev	GGGGACCACTTTGTACAAGAAAGCTGGGTACTAACTGCGTATACACATCAG CACACTG
GW-CENP-C-1002-1093-Fw (FBD)	GGGGACAAGTTTGTACAAAAAAGCAGGCTTAATTGATCAAAACTCAGATGA TGTT
GW-CENP-C-1002-1093-Rev (FBD)	GGGGACCACTTTGTACAAGAAAGCTGGGTATTAAGTATTTAGTTCCTCAGCT TCCACC
GW-CENP-C-1202-1411-Fw	GGGGACAAGTTTGTACAAAAAAGCAGGCTTAATGGAGGCTGAGAAGGTGCC CA
GW-CENP-C-1202-1411-Rev	GGGGACCACTTTGTACAAGAAAGCTGGGTACTAACTGCGTATACACATCAG CACACTG
**Conventional cloning:**	
CENP-C-NotI-Fw	ATAAGAAGCGGCCGCATGTCGAAGCCCCAGAACAACGAC

CENP-C-XbaI-Rev	GCTCTAGAACTGCGTATACACATCAGCACACT
**Mutagenesis:**	
PP4c-D85N-Fw	GGGCGATTTCGTGAACCGCGGCTACTATAGTG
PP4c-D85N-Rev	CACTATAGTAGCCGCGGTTCACGAAATCGCCC
PP4c-H115N-Fw	CTGATCCGGGGCAACAACGAGTCGCGCCAAATC
PP4c-H115N-Rev	GATTTGGCGCGACTCGTTGTTGCCCCGGATCAG
CENP-C-Δ1048-1066-Fw (ΔFIM)	TCAGAACATGTCAAAGAGTCGTCCCGCGAAATCAAAAAAAGGGAAGTCTG
CENP-C-Δ1048-1066-Rev (ΔFIM)	CAGACTTCCCTTTTTTTGATTTCGCGGGACGACTCTTTGACATGTTCTGA
**IVTT PCR products:**	
5'-T7-C1	GAATTAATACGACTCACTATAGGGAGAGCCGCCACCATGCCACCTCCTATCGAATATGTTG
3'-C1	TTACGGAGGAGTGGGTGTGTTAACGCGAAG
5'-T7-C2	GAATTAATACGACTCACTATAGGGAGAGCCGCCACCATGATTGATCAAAACTCAGATGATG
3'-C2	TTATGCTTGCACTTCAGTTTCTTCCCACGT
5'-T7-C3	GAATTAATACGACTCACTATAGGGAGAGCCGCCACCATGGAGGCTGAGAAGGTGCCCAAGA
3'-C3	TTAACTGCGTATACACATCAGCACACTGAC
5'-T7-C4	GAATTAATACGACTCACTATAGGGAGAGCCGCCACCATGGAGGAATCTCAAAATAAAAATG

3'-C4	TTATGATCTCCGAATACCGGTAGTATTTAG
5'-T7-C5	GAATTAATACGACTCACTATAGGGAGAGCCGCCACCATGAAGCGCGGTCAAGTTCCACTTC
3'-C5	TTAACTGGCGTTTTCATCCGACATACTTGC
5'-T7-C6	GAATTAATACGACTCACTATAGGGAGAGCCGCCACCATGATTGATCAAAACTCAGATGATG
3'-C6	TTATGATCTCCGAATACCGGTAGTATTTAG
5'-T7-C7	GAATTAATACGACTCACTATAGGGAGAGCCGCCACCATGAAGCGCGGTCAAGTTCCACTTC
3'-C7	TTATGCTTGCACTTCAGTTTCTTCCCACGT
5'-T7-C8	GAATTAATACGACTCACTATAGGGAGAGCCGCCACCATGATTGATCAAAACTCAGATGATG
3'-C8	TTAGTTAAACTTGCTAGGATCCATGGTGTGA
5'-T7-C9	GAATTAATACGACTCACTATAGGGAGAGCCGCCACCATGATTGATCAAAACTCAGATGATG
3'-C9	TTAAGTATTTAGTTCCTCAGCTTCCACCGGC
5'-T7-C10	GAATTAATACGACTCACTATAGGGAGAGCCGCCACCATGACTACCGGTATTCGGAGATCAA
3'-C10	TTATGCTTGCACTTCAGTTTCTTCCCACGT
5'-T7-C11	GAATTAATACGACTCACTATAGGGAGAGCCGCCACCATGTTTATGAGTGGCTTCATAGAGC
3'-C11	TTATGCTTGCACTTCAGTTTCTTCCCACGT
5'-T7-Flfl-1-168	GAATTAATACGACTCACTATAGGGAGAGCCGCCACCATGACGACTGACACCCGCCGACGCG
3'-Flfl-1-168	TCATTACTATCACTCCAGAGCCATCGACAACT

**DUET cloning:**	
Falafel-NdeI-Fw	ATACATATGACGACTGACACCCGCCGACGC
Falafel-NdeI-Rev	TATCATATGCTATGCCTGACGCGCGCTTTT
TEV::Flfl-1-168-Fw	AAGAATTCAGAAAACCTGTATTTTCAGGGCACGACTGACACCCGCCGACG
TEV::Flfl-1-168-Rev	TTTGCGGCCGCCTACTCCAGAGCCATCGACAAC
TEV::Flfl-1-122-Fw	AAGAATTCAGAAAACCTGTATTTTCAGGGCACGACTGACACCCGCCGACG
TEV::Flfl-1-122-Rev	TTTGCGGCCGCCTACCGCTCATCCTCAGACT
PP4c-NdeI-Fw	ATACATATGTCCGACTACAGCGACCTGGAC
PP4c-XhoI-Rev	TACTCGAGTTAGAGAAAGTAGTCCGCCTGAGG
R2-NdeI-Fw	ACGCATATGGTCACCATGGAAAACAGCGAC
R2-XhoI-Rev	TACTCGAGCTACTGCATCATCACCTCCTGGCT
TEV::CENP-C-1002-1093-Fw (FBD)	AAGAATTCAGAAAACCTGTATTTTCAGGGCATTGATCAAAACTCAGATGA
TEV::CENP-C-1002-1093-Rev (FBD)	CTAGCGGCCGCTTAAGTATTTAGTTCCTCAGCTT
**dsRNA preparation**	
KAN-dsRNA-Fw	TAATACGACTCACTATAGGGAGAGACAATCTATCGCTTGTATG
KAN-dsRNA-Rev	TAATACGACTCACTATAGGGAGAGGAATCGAATGCAACCGGCGC
Falafel-dsRNA1-Fw	TAATACGACTCACTATAGGGAGAATGACGACTGACACCCGC

Falafel-dsRNA1-Rev	TAATACGACTCACTATAGGGAGAACAACTTTTCCTTTCGCA
Falafel-dsRNA2-Fw	TAATACGACTCACTATAGGGAGACAAGAAGACCAAATCGGCCTC
Falafel-dsRNA2-Rev	TAATACGACTCACTATAGGGAGAGTAATCGCAGAGCGCCGAGGA
Falafel-dsRNA3-Fw	TAATACGACTCACTATAGGGAGAAACGCTCTAGCGTCGGTCCGC
Falafel-dsRNA3-Rev	TAATACGACTCACTATAGGGAGAGATGATGTTCCTCCTTGCTGTGC
PP4c-dsRNA1-Fw	TAATACGACTCACTATAGGGAGAATGTCCGACTACAGCGAC
PP4c-dsRNA1-Rev	TAATACGACTCACTATAGGGAGACCACACGGCCGTCGATCC
PP4c-dsRNA2-Fw	TAATACGACTCACTATAGGGAGACGCAAGTAGCCAATCCAGCTC
PP4c-dsRNA2-Rev	TAATACGACTCACTATAGGGAGAATCTTCACCCGGAGGATGGC
CENP-C-dsRNA1-Fw	TAATACGACTCACTATAGGGAGACTTCGCGTTAACACACCCACT
CENP-C-dsRNA1-rev	TAATACGACTCACTATAGGGAGACCTGGCAGCTTTTCAGAAATT
CENP-C-dsRNA2-Fw	TAATACGACTCACTATAGGGAGAGGTACCACCTCCTATCGAATA
CENP-C-dsRNA2-Rev	TAATACGACTCACTATAGGGAGAGAATTCCAATTTGGATCTGGA
mts-dsRNA-Fw	TAATACGACTCACTATAGGGAGAGGCAGTCTTTCCCTTCGTATATC
mts-dsRNA-Rev	TAATACGACTCACTATAGGGAGACGAACTTGTGTCTCTGTCAACTG

SUPPLEMENTARY REFERENCES

1. Myles T, Schmidt K, Evans DR, Cron P, Hemmings BA. Active-site mutations impairing the catalytic function of the catalytic subunit of human protein phosphatase 2A permit baculovirus-mediated overexpression in insect cells. *Biochem J* **357**, 225-232 (2001).
